# Activity-Based Profiling of Papain-like Cysteine Proteases During Late-Stage Leaf Senescence in Barley

**DOI:** 10.3390/plants14203132

**Published:** 2025-10-11

**Authors:** Igor A. Schepetkin, Andreas M. Fischer

**Affiliations:** Department of Plant Sciences and Plant Pathology, Montana State University, Bozeman, MT 59717, USA

**Keywords:** activity-based proteomics, *Hordeum vulgare* L., leaf senescence, papain-like cysteine protease, phylogenetic tree analysis, tandem mass spectrometry

## Abstract

Leaf senescence is a developmental process that allows nutrients to be remobilized and transported to sink organs. Previously, papain-like cysteine proteases (PLCPs) have been found to be highly expressed during leaf senescence in different plant species. In this study, we analyzed active PLCPs in barley (*Hordeum vulgare* L.) leaves during the terminal stage of natural senescence. Anion exchange chromatography of protein extracts from barley leaves, harvested six weeks after anthesis, followed by activity assays using the substrates Z-FR-AMC and Z-RR-AMC, revealed a single prominent peak corresponding to active PLCPs. This hydrolytic activity was completely inhibited by E-64, a potent and irreversible inhibitor of cysteine proteases. Fractions enriched for PLCP activity were affinity-labeled with DCG-04 and subjected to SDS-PAGE fractionation, separating two major bands at 43 and 38 kDa. These bands were analyzed using tandem mass spectrometry, allowing the identification of eleven PLCPs. Identified enzymes belong to eight PLCP subfamilies, including CTB/cathepsin B-like (*Hv*Pap-19 and -20), RD19/cathepsin F-like (*Hv*Pap-1), ALP/cathepsin H-like (*Hv*Pap-12 or aleurain), SAG12/cathepsin L-like A (*Hv*Pap-17), CEP/cathepsin L-like B (*Hv*Pap-14), RD21/cathepsin L-like D (*Hv*Pap-6 and -7), cathepsin L-like E (*Hv*Pap-13 and -16), and XBCP3 (*Hv*Pap-8). Among the identified PLCPs, *Hv*Pap-6 was the most abundant. Peptides corresponding to *Hv*Pap-6 were identified in both the 43 kDa and 38 kDa bands in approximately the same quantity based on total spectral count. Thus, our results indicate that two active *Hv*Pap-6 isoforms can be isolated from barley leaves at late senescence.

## 1. Introduction

Barley is a global crop, ranking fourth among cereals in the world in terms of production after maize, rice, and wheat [1]. It is a versatile crop used for human food, animal feed, and for malting, especially in the brewing industry [2].

During leaf senescence, nutrients are mobilized and translocated from the senescing tissues to young leaves, storage organs, or developing seeds [3,4,5]. During this process, protein breakdown involves several classes of proteases, including serine proteases, metalloproteases, aspartic proteases, cysteine proteases, and the proteasome [6,7]. Among these enzymes, cysteine proteases are the most strongly associated with leaf senescence in various species [8]. Papain-like cysteine proteases (PLCPs; family C1A in the MEROPS classification system) are one of the most abundant groups of cysteine proteases [9,10]. These enzymes are synthesized in an inactive form to prevent their premature activity. This inactive state is achieved by a prodomain, an N-terminal extension that blocks the active site and prevents binding with substrates [11]. The protease domain contains in its active center a nucleophilic cysteine residue which, in combination with histidine and aspartic acid, forms a catalytic triad [9].

In plants, PLCPs play a crucial role in nitrogen remobilization, programmed cell death, plant immunity, and the initiation of signaling pathways [8,12,13,14,15]. They are involved in breaking down proteins in senescing leaves, releasing amino acids for translocation to other parts of the plant where they are needed, such as for seed filling [16,17,18]. During plant senescence, high expression was found for many *PLCP* genes in different plant species [19,20]. In barley, PLCPs have been classified as *Hv*Pap-1 to *Hv*Pap-42 [8]. Significant upregulation of *HvPap* genes (including *HvPap*-*1*, *2*, *4*, *6-8*, *12-15*, *17*, *19*, *20*, and *22*) has been previously reported during barley leaf senescence [8,21,22,23,24]. Understanding the functional roles of specific PLCPs during leaf senescence provides foundational knowledge for the improvement of crop traits, including seed/grain protein content and nitrogen use efficiency [25,26,27]. The overlapping activities and functional redundancy of PLCPs make it difficult to isolate and study the contribution of specific proteases to the overall process of plant senescence [28,29]. On the other hand, gene expression profiles do not always correspond to protein-level changes and do not reflect many functional features of proteomes [29].

Mass spectrometry (MS) is a powerful tool that can assist breeding aimed at developing superior barley varieties [30]. Counting of peptide spectra from MS datasets allows for label-free quantification, where the number of spectra assigned to a protein is used as an indicator of its relative abundance [31,32,33]. Combining activity-based protein profiling (ABPP) with tandem MS analysis is a powerful approach for studying protease function [34].

We have previously characterized PLCP activities at different stages of barley leaf senescence, using fluorogenic substrates, specific inhibitors, DCG-04 labeling, and immunoblotting [35]. Since PLCP activities are maximal during late senescence, while ribulose-1,5-bisphosphate carboxylase/oxygenase (Rubisco) is largely depleted [35] (hence, not a problem for tandem MS identification of low-abundance proteins), we chose that developmental stage to identify PLCPs critical for natural barley leaf senescence.

## 2. Results and Discussion

### 2.1. Isolation of PLCPs Using Ion-Exchange and Affinity Chromatography

**Figure 1:**
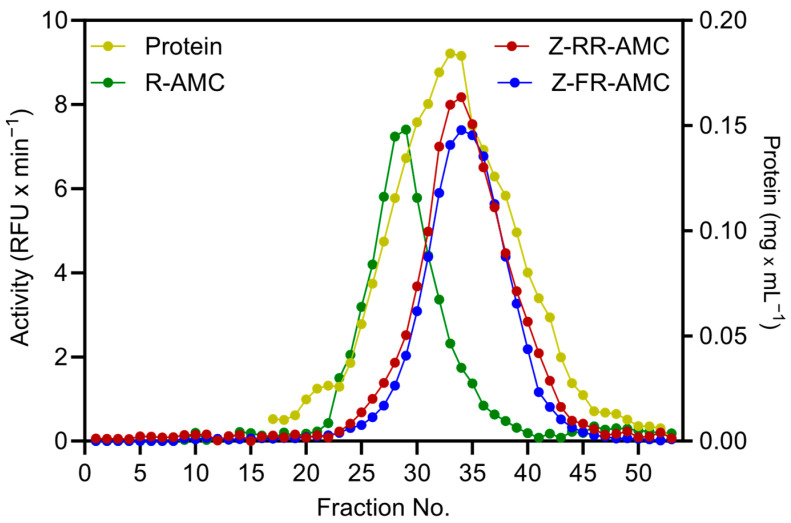
Anion exchange chromatography of late-senescence barley leaf PLCPs. Fractions eluted with a gradient of NaCl (0.0 to 1.0 M NaCl) from DEAE-Sepharose were tested for PLCP activity using 25 µM of the substrates Z-FR-AMC (blue line) and Z-RR-AMC (dark red line); aminopeptidase activity was tested using 25 µM of the substrate R-AMC (green line). Total protein (yellow line) was determined using Bradford reagent. Abbreviation: RFU, relative fluorescence units.

Previous studies showed that cysteine proteases can be purified from plant protein extracts by anion-exchange chromatography (e.g., [36,37,38]). In the present study, the total protein extract from fully senesced leaves collected from barley shoots at 6 weeks after anthesis was loaded on a DEAE-Sepharose column, followed by washing with loading buffer. When a linear gradient of NaCl (0.0–1.0 M) was used with the elution buffer, a single peak of enzyme activity was identified, based on assays performed with the fluorogenic substrates Z-FR-AMC and Z-RR-AMC (Figure 1).

The activity of the peak fractions measured with these substrates was completely inhibited by E-64, a specific inhibitor of cysteine proteases [39]. The R-AMC cleaving aminopeptidase activity eluted earlier than the Z-FR-AMC/Z-RR-AMC cleaving activities and was insensitive to E-64 even at high concentration (up to 25 µM), but was inhibited by bestatin, an aminopeptidase inhibitor, and 1,10-phenanthroline, a metallopeptidase inhibitor (Table 1).

Because the activity of aleurain (a cysteine aminopeptidase) was inhibited by E-64 [40], we suggest that barley PLCPs can be enriched in one step using DEAE-Sepharose. It should be noted that fractionation of PLCPs from maize leaves by ion-exchange chromatography also produced a single Z-FR-AMC cleavage activity peak, which is sensitive to E-64 inhibition [37].

The active fractions (based on the Z-FR-AMC/Z-RR-AMC enzymatic assays) were pooled and labeled with DCG-04, a biotinylated epoxide probe that binds covalently and irreversibly to the active site of PLCPs [41]. This probe has previously been used to identify cysteine proteases involved in senescence and abiotic stress responses in maize, wheat, and other plants [37,42,43].

The labeled proteins were captured on streptavidin beads; the column was washed with 1% SDS/1% NP-40 and eluted with Laemmli reducing sample buffer with excess biotin (25 mM) and heat [44]. Proteins were separated by SDS-PAGE. The protein bands, visualized by Coomassie Blue staining, showed that DCG-04-labeled proteases are separated into two bands with molecular weights of 43 and 38 kDa, similar to those found in our previous experiments using SDS-PAGE and streptavidin-horseradish peroxidase to detect DCG-04-labeled barley proteases [35]. A typical SDS-PAGE gel image with these two bands is shown in the Supplementary Materials (Figure S1). The bands were then excised and digested with trypsin, followed by peptide extraction and analysis by tandem MS.

### 2.2. Semiquantitative Tandem MS Analysis of Barley PLCPs

**Figure 2:**
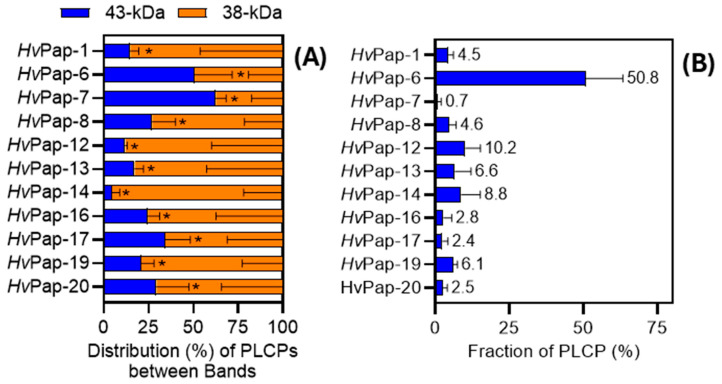
Tandem MS analysis of late-senescence barley leaf PLCPs. The distribution (in %) of PLCPs between gel bands with molecular masses of 38 and 43 kDa, based on total spectral counts, is presented in panel (**A**), while panel (**B**) shows the relative abundance (in %) of the identified PLCPs (based on both gel bands). In both panels, the data represent the mean value ± S.D. of three independent experiments. Statistically significant differences (* *p* < 0.05) by *t*-test in the distribution of PLCPs between the two gel bands are shown in panel (**A**).

Based on tandem MS analysis [32], eleven PLCPs, including *Hv*Pap-1, -6, -7, -8, -12, -13, -14, -16, -17, -19, and -20, were identified in the protein extract from barley leaves harvested six weeks after anthesis (Figure 2). Total spectral counts for all identified PLCPs from one representative experiment are shown in Table 2. In addition, Supplementary Tables S1–S11 provide MS data showing peptide identification, their Mascot ion scores, as well as m/z (mass-to-charge ratio) and charge data for selected samples from one representative experiment. Four active PLCPs, including *Hv*Pap-6, -12, -13, and -14, have been previously identified in senescing barley leaves, although without semi-quantitative tandem MS analysis based on total spectral count [45].

Peptides corresponding to *Hv*Pap-6 and *Hv*Pap-7 were identified in both 43 kDa and 38 kDa gel bands in approximately the same quantity based on total spectral counts (Figure 2A). The total number of peptide spectra associated with each PLCP in both gel bands was calculated, and their proportion among all eleven identified PLCPs was estimated. Based on this analysis, *Hv*Pap-6 is the most abundant PLCP in late-senescence barley leaves (Figure 2B). Using immunoblotting, Cohen et al. [23] also found a significant increase in *Hv*Pap-6 protein levels during barley leaf senescence. Our findings are consistent with recent results by Havé et al. [43] showing that triticain α, an ortholog of *Hv*Pap-6, is the major active PLCP in naturally senescent wheat leaves. Furthermore, Sekhon et al. [46] demonstrated the functional importance of Mir3 (a maize *Hv*Pap-6 ortholog) for leaf senescence, using a genome-wide association study (GWAS), gene expression, and protease activity assays. Those authors also showed that knockout of *Arabidopsis* RD21A, an ortholog of *Hv*Pap-6, triticain α, and Mir3, leads to a delayed-senescence phenotype. A delay of whole-plant senescence was also demonstrated by Pružinská et al. [47] in *rd21a*/*aalp* (cathepsin H) double mutants, but not in *rd21a* (single) mutants. Thus, evidence from several plant species, including barley, strongly points to the functional relevance of RD21-like PLCPs for leaf senescence, but their participation in protein degradation/nitrogen remobilization, senescence regulation, or biotic/abiotic stress tolerance of senescing tissues remains to be established. Phylogenetic relationships between PLCPs are discussed in the next section.

**Figure 3:**
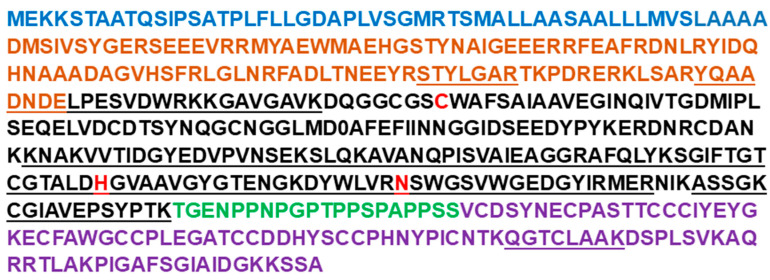
Primary full-length sequence of the *Hv*Pap-6 protein identified by tandem MS as the predominant active PLCP during late-stage leaf senescence in barley. Peptide sequences detected by tandem MS are underlined. The amino acid coverage is based on 3 independent samples. Amino acids marked in blue belong to the signal peptide; brown—inhibitor domain; black—protease domain; green—proline-rich domain; violet—granulin domain; catalytic residues (C, H, N) are shown in red. The amino acids were attributed to the different domains based on [48].

In *Arabidopsis*, RD21A is known to exist as two forms, an intermediate form containing the C-terminal granulin domain and a mature form from which the granulin domain has been removed [48]. Our results suggest that *Hv*Pap-6 could also be heterogeneous, which explains the presence of active isoforms differing by approximately 5 kDa (molecular weights of ~43 and 38 kDa). Thus, the 43 kDa band is likely to be a longer-chain form of *Hv*Pap-6, implying partial cleavage of the prodomain and/or granulin domain. Indeed, although the STYLGAR peptide from the prodomain of *Hv*Pap-6 was detected only once in the 43 kDa band with a relatively low peptide identification probability (19%), another peptide (YQAADNDELPESVDWR), which covers the end of the prodomain and the beginning of the protease domain, was detected multiple times with a high peptide identification probability (91–100%). Thus, *Hv*Pap-6 may require only partial cleavage within the prodomain for activation. On the other hand, the granulin peptide QGTCLAAK of *Hv*Pap-6 was detected in both 43 kDa and 38 kDa bands with high probability (99%) (Figure 3).

It should be noted that no peptides were detected that cover a conserved region around the catalytic cysteine. This was found previously in other studies on cysteine proteases using DCG-04-based proteomics (e.g., [29]) and may be explained by the fact that irreversible binding of DCG-04 to the catalytic cysteine changes the molecular weight of peptide ions. However, because this conserved region exhibits high amino acid sequence similarity across PLCPs, its identification by MS analysis provides limited value in distinguishing closely related proteins [49].

### 2.3. Phylogenetic Analysis of Barley PLCPs

Previously, 42 barley PLCPs (designated *Hv*Pap-1 to *Hv*Pap-42) have been reported and classified into 8 subfamilies, including cathepsin B-like, cathepsin F-like, cathepsin H-like, and 5 subgroups (from A to E) of cathepsin L-like [8]. To identify previously not characterized barley PLCPs, we explored the UniProt database (https://www.uniprot.org) and found 64 barley PLCPs, including 22 proteases with identity/similarity < 90% as compared to the previously described *Hv*Paps (Table 3). To gain insights into the subfamily classification of the barley PLCPs and to investigate the relationship of new members with known PLCPs, we constructed a phylogenetic tree using these 64 barley PLCPs, together with 32 PLCPs from *Arabidopsis thaliana* and four PLCPs from other plant species to increase the number of members of the XBCP3 and THI1 groups. PLCPs with >90% identity/similarity in comparison with sequences of the previously reported barley PLCPs were not included in the analysis because (a) they were considered to represent minor genetic variations based on the pangenome concept [50] and (b) PLCPs with high identity/similarity will not form branches on the phylogenetic tree, creating noise that can complicate the analysis.

**Figure 4:**
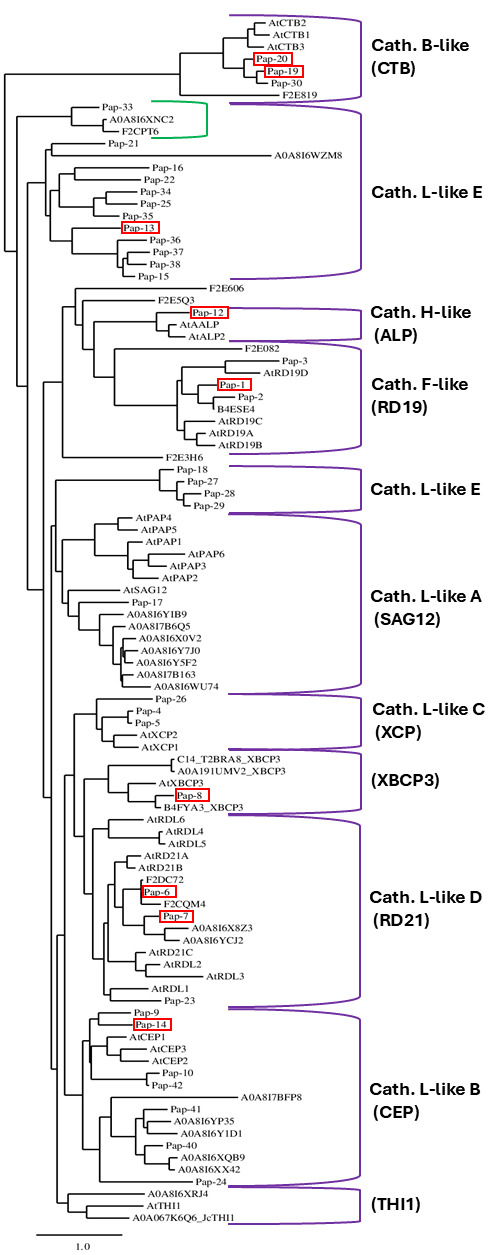
Phylogenetic tree of barley PLCPs in which proteins are clustered based on full-length amino acid sequence comparisons. PLCPs present in late-senescence barley leaves, as identified by tandem MS in the present study, are indicated by red rectangles. The green bracket denotes a distinct subcluster within the cathepsin L-like E subfamily.

As a result, the PLCPs were clustered into ten subfamilies (Figure 4). Most barley PLCPs could be classified in the subfamilies described by Richau et al. [13] and/or Díaz-Mendoza et al. [8]. The exceptions are *Hv*Pap-31, -32, and -39, which were not included in any subfamilies, confirming the previous phylogenetic tree analysis [8]. Although Diaz-Mendoza et al. [8] did not classify *Hv*Pap-24 as belonging to any cluster, our analysis suggests that this protease belongs to the L-like B subfamily (CEP cluster according to Richau et al. [13]). One barley PLCP (UniProt ID: A0A8I6XRJ4) is represented in the THI1 subfamily together with protease AtTHI1 from *A. thaliana* (UniProt ID: Q9LNC1) and one member from *Jatropha curcas* (UniProt ID: A0A067K6Q6). Based on the present analysis and the phylogenetic tree analysis by Liu et al. [54], *Hv*Pap-8 is located in the XBCP3 cluster, not in the cathepsin L-like D subfamily (RD21 cluster) as previously reported [8]. Both the RD21 and XBCP3 clusters include PLCPs with a granulin domain, although proteases without the granulin domain could also be present in these subfamilies [37].

The eleven PLCPs identified by tandem MS analysis in the present study are scattered across eight PLCP subfamilies (indicated by red rectangles in Figure 4), including CTB/cathepsin B-like (*Hv*Pap-19 and -20), RD19/cathepsin F-like (*Hv*Pap-1), ALP/cathepsin H-like (*Hv*Pap-12 or aleurain), SAG12/cathepsin L-like A (*Hv*Pap-17), CEP/cathepsin L-like B (*Hv*Pap-14), RD21/cathepsin L-like D (*Hv*Pap-6 and -7), cathepsin L-like E (*Hv*Pap-13 and -16), and XBCP3 (*Hv*Pap-8). As *Hv*Pap-6 was the most prominent PLCP identified in this study and is located in the RD21/cathepsin L-like D cluster, Table 4 specifically compares *Hv*Pap-6 and *Hv*Pap-7 with orthologs from wheat (triticain α), maize (Mir3), and *Arabidopsis* (RD21A). All these proteases are active in senescing leaves, and for two of them (RD21A and Mir3), available data point to a functional involvement in the senescence process [46,47].

Based on previously reported PLCP classifications by [8,13,37] and the present work, the distribution of PLCPs into ten subfamilies for *H. vulgare*, *A. thaliana*, and *Zea mays* is shown in Table 5. It should be noted that 18 barley PLCPs belong to the cathepsin L-like E subfamily, forming the largest cluster, which has no related members in *Arabidopsis* and maize.

To find orthologs of the cathepsin L-like E family members in other plant species, we extracted proteins with similarity > 50% to these eighteen barley PLCPs from the UniProt database and found 326 orthologs. All identified genes except one (A0A8J5GYP2) belong to the Poaceae family, mainly to the BOP clade (Bambusoideae, Oryzoideae, and Pooideae), and have no related family members in maize and sorghum (see Supplementary Table S12), suggesting that cathepsin L-like E proteases are mostly restricted to the BOP clade of the Poaceae family with the C3 photosynthetic pathway.

Using tandem MS analysis, we identified only two barley proteases from the cathepsin L-like E subfamily, *Hv*Pap-13 and *Hv*Pap-16, with relatively low abundance (Figure 2B). Thus, PLCPs of this subfamily may be more important at other stages of plant development. Moreover, organ-specific PLCP activity has recently been found in maize roots [37]. Some PLCPs also play a crucial role in hydrolyzing storage proteins during seed germination [55,56]. Further studies are needed to identify cathepsin L-like E proteases at different stages of leaf development as well as in different plant organs, such as during seed germination or root development.

## 3. Materials and Methods

### 3.1. Materials and Reagents

E-64 (trans-epoxysuccinyl-L-leucylamido-(4-guanidino)butane), Z-Phe-Arg-7-amino-4-methylcoumarin (Z-FR-AMC), 1,10-phenanthroline, bestatin, DEAE-Sepharose CL-6B, and dithiothreitol (DTT) were from Millipore-Sigma (St. Louis, MO, USA). CAA0225 was from EMD Millipore Corporation (Burlington, MA, USA). A color-coded prestained protein marker was obtained from Cell Signaling Technologies (Danvers, MA, USA). Z-Arg-Arg-7-amino-4-methylcoumarin (Z-RR-AMC) was from Echelon Biosciences (Salt Lake City, UT, USA), and Arg-7-amido-4-methylcoumarin (R-AMC) was obtained from AK Scientific (Palo Alto, CA, USA). Dimethyl sulfoxide (DMSO) was from Acros Organics (Fair Lawn, NJ, USA). DCG-04, a biotinylated E-64 derivative [41], was obtained from Psyclo Peptide Inc. (Shanghai, China). High-capacity streptavidin agarose resin was from Thermo Scientific (Rockford, IL, USA).

### 3.2. Plant Material

Barley plants (*Hordeum vulgare* L. var. ‘GemCraft’) [57] were grown as described previously [35]. Briefly, barley plants (three plants per 4 L pot) were grown in potting soil in a greenhouse bay of the Plant Growth Center at Montana State University (Bozeman, MT, USA) between August and November 2023, with a 22 °C/18 °C day/night temperature cycle. Days were extended to a 16 h photoperiod using Son-Agro 430 W high-pressure sodium lamps (Philips, Somerset, NJ, USA). The photosynthetic photon flux density (PPFD) from artificial light was 120–180 μmol m^−2^ s^−1^ and 600–800 μmol m^−2^ s^−1^ from natural light at the leaf level. Plants were fertilized once per week until flowering with Peter’s Professional General-Purpose fertilizer (250 mL per pot; 4 g/L; Scotts-Sierra Horticultural Products Company, Marysville, OH, USA). Fully senesced leaves from 40 to 50 plants were collected six weeks after anthesis and stored at −20 °C until protein extraction.

### 3.3. Ion-Exchange and Affinity Purification of PLCPs

Soluble proteins from barley leaves were extracted by homogenizing 10 g of leaf powder in 200 mL of ice-cold 50 mM Tris-HCl buffer (pH 7.4). Homogenates were filtered through one layer of Miracloth (Calbiochem, San Diego, CA, USA), centrifuged at 20,000× *g* for 20 min at 4 °C, and the supernatants were then filtered through a 0.45 μm filter (Avantor, Radnor, PA, USA). PLCPs were separated using anion-exchange chromatography on a DEAE-Sepharose CL-6B column (volume 6 mL) equilibrated with 0.05 M Tris-HCl buffer (pH 7.4). The column was washed with equilibration buffer, and bound material was eluted with a NaCl gradient (0.0–1.0 M). Collected fractions (1 mL each) were kept in an ice bath and used for measurements of peptidase activity and protein content. Fractions with active cysteine proteases were pooled. To reduce the NaCl concentration and exchange the buffer to 0.1 M Na-citrate (pH 5.5), three sequential filtration steps using 10 kDa ultrafiltration 15 mL units (Merck Millipore Ltd., Carrigtwohill, County Cork, Ireland) were utilized, and the samples were incubated with gentle shaking with 10 µM DCG-04 in the presence of 2 mM DTT for 3 h at 37 °C. No remaining PLCP activity was found after three hours, as confirmed using Z-FR-AMC and Z-RR-AMC fluorogenic substrates. The intensity of DCG-04-labeled barley cysteine proteases was determined in our previous experiments using streptavidin-horseradish peroxidase [35]. After the incubation, proteins were rapidly precipitated using 80% acetone under inclusion of 100 mM NaCl [58].

Affinity enrichment of DCG-04-reactive PLCPs was performed using streptavidin-agarose. Each sample was incubated with 150 μL bed volume streptavidin-agarose for 60 min at room temperature. To reduce nonspecific binding, the column was washed with 1% SDS and 1% NP-40 in 0.05 M Tris-HCl buffer (pH 7.4). After washing, bound polypeptides were eluted by adding 100 µL of Laemmli reducing sample buffer with excess biotin (25 mM) and boiling for 7 min [44].

DCG-04-reactive proteins were then separated on ready-made ExpressPlusTM 4–12% acrylamide gels (GenScript Inc., Piscataway, NJ, USA) in Tris-MOPS SDS-PAGE Running Buffer (GenScript). The gels were fixed for 45 min, stained with Coomassie Brilliant Blue R-250 overnight, and destained with 50% methanol, 10% acetic acid, and 40% water. Visible bands from the Coomassie-stained gels were excised and shipped to the IDeA National Resource for Quantitative Proteomics at the University of Arkansas (Little Rock, AR, USA).

### 3.4. Enzymatic Assays and Protein Determination

Fluorogenic substrates Z-FR-AMC to determine cathepsin B- and L-like activity, and Z-RR-AMC to determine cathepsin B-like activity, were used. These substrates show fluorescence when AMC is released as a consequence of the hydrolysis of the Arg-AMC bond. The emitted fluorescence was detected with a SpectraMax M2 microplate reader (Molecular Devices, San Jose, CA, USA) with λ_ex_ = 360 nm and λ_em_ = 460 nm. Before use, the substrates were dissolved in DMSO at 10 mM and stored at −20 °C. The measurements were made in 96-well black microplates (PerkinElmer Inc., Waltham, MA, USA), and each well contained a 100 μL final volume of 0.1 M Na-citrate buffer (pH 5.5) with DTT (2 mM), an aliquot of extract or chromatographic fraction, and fluorogenic substrate (25 μM). The reaction was initiated by the addition of the substrate. The final concentration of DMSO in microplate wells was 1% in all assays. The assays were conducted at room temperature, and relative fluorescence readings were recorded over a period of 10 min.

For the inhibition assays, an inhibitor (1 µL of stock solution of E-64 or bestatin in DMSO, or stock solution of 1,10-phenanthroline in ethanol) at different concentrations was added to the reaction mixture. For all inhibitors tested, the concentration of an inhibitor that caused 50% inhibition of the enzymatic reaction (IC_50_) was calculated by plotting percent inhibition against the logarithm of inhibitor concentration (at least six data points). The data are presented as the mean values of at least three independent experiments.

Protein concentration was determined using Bradford reagent (Thermo Scientific, Rockford, IL, USA) and bovine serum albumin as a standard.

### 3.5. Gel-Based Tandem MS Analysis

Each SDS-PAGE gel band was subjected to in-gel trypsin digestion [59]. Gel segments were destained in 50% methanol with 50 mM ammonium bicarbonate, followed by reduction in 10 mM Tris[2-carboxyethyl]phosphine (Pierce, Rockford, IL, USA) and alkylation in 50 mM iodoacetamide (Sigma-Aldrich, St. Louis, MO, USA). Gel slices were then dehydrated in acetonitrile (Thermo Fisher Scientific, Rockford, IL, USA), followed by the addition of 100 ng porcine sequencing grade modified trypsin (Promega, Madison, WI, USA) in 50 mM ammonium bicarbonate (Sigma-Aldrich) and incubation at 37 °C for 12–16 h. Peptide products were then acidified in 0.1% formic acid (Pierce).

Tryptic peptides were separated by reverse phase XSelect CSH C18 2.5 µm resin (Waters, Milford, MA, USA) on an in-line 150 × 0.075 mm column using a nanoAcquity UPLC system (Waters). Peptides were eluted using a 60 min gradient from 98:2 to 65:35 buffer A–B ratio (buffer A = 0.1% formic acid, 0.5% acetonitrile; buffer B = 0.1% formic acid, 99.9% acetonitrile). Eluted peptides were ionized by electrospray (2.4 kV) followed by tandem MS analysis using higher-energy collisional dissociation (HCD) on an Orbitrap Fusion Tribrid mass spectrometer (Thermo Fisher Scientific) in top-speed data-dependent mode. MS data were acquired using the FTMS analyzer in profile mode at a resolution of 240,000 over a range of 375 to 1500 m/z. Following HCD activation, tandem MS data were acquired using the ion trap analyzer in centroid mode and normal mass range with precursor mass-dependent normalized collision energy between 28.0 and 31.0.

### 3.6. Database Searching and Criteria for Protein Identification

Proteins were identified by database search using Mascot (Matrix Science, London, UK; version 2.6.2) with a parent ion tolerance of 3 ppm and a fragment ion tolerance of 0.5 Da. Mascot was set up to search the 2024_06 UniProt *Hordeum_vulgare* database (34,528 entries), assuming the digestion enzyme trypsin. Scaffold (version 5.3.3, Proteome Software Inc., Portland, OR, USA) was used to validate tandem MS-based peptide and protein identifications. Peptide identifications were accepted if they could be established at greater than 6.0% probability to achieve a false discovery rate (FDR) < 1.0% by the Peptide Prophet algorithm [60] with Scaffold delta-mass correction. Protein identifications were accepted if they could be established at >92.0% probability to achieve an FDR < 1.0% and contained at least two identified peptides. Protein probabilities were assigned by the Protein Prophet algorithm [61].

### 3.7. Computational Methods

For the generation of a phylogenetic tree, 64 barley PLCP amino acid sequences were retrieved from the UniProt open-access resource [51,52] were used. Additionally, 32 PLCPs of *A. thaliana* were included [13], as well as three members of the XBCP3 subfamily from other plant species (T2BRA8, a cysteine protease CP14 from *Nicotiana tabacum*; A0A191UMV2, a PLCP from *Nicotiana benthamiana*; B4FYA3, a xylem bark cysteine peptidase 3 from *Zea mays*), and one member of the THI1 subfamily from *Jatropha curcas* (A0A067K6Q6). The Phylogeny.fr platform was used for the construction of the tree [62].

## 4. Conclusions

In conclusion, eleven active PLCPs were identified in protein extracts from late-senescence barley leaves using tandem MS analysis. The identified PLCPs were distributed across eight PLCP subfamilies. Among the identified PLCPs, *Hv*Pap-6 was the most abundant and was present in barley leaves in at least two active isoforms. This finding may facilitate functional comparisons with PLCPs from other plant species and will contribute to the elucidation of their specific roles during barley leaf senescence. Results obtained by other researchers in *Arabidopsis*, wheat, and maize suggest that *Hv*Pap-6 and its orthologs (RD21-like proteases) are functionally important during leaf senescence, but their exact role in nitrogen remobilization, senescence regulation, and/or abiotic/biotic stress tolerance of senescing tissues remains to be established using transgenic approaches or profiting from natural variation. Exploration of the barley pangenome (https://panbarlex.ipk-gatersleben.de/; [53]) suggests the absence of *Hv*Pap-6 from one accession (HOR 7385), providing an entry point for *Hv*Pap-6 functional studies.

Further studies are needed to fully establish the classification of barley PLCPs, especially with respect to the cathepsin L-like E subfamily.

## Figures and Tables

**Table 1:** Effect of protease inhibitors on cleavage of Z-RR-AMC, Z-FR-AMC, and R-AMC substrates by fractions obtained by ion-exchange chromatography from barley leaf extract. Extracts were obtained from senescing leaves collected six weeks after anthesis.

Fraction	Substrate	Effect/IC_50_
E-64		
Fr. No 26–27	R-AMC	No inhibition up to 25 µM
Fr. No 35–36	Z-RR-AMC	9.1 ± 2.2 nM
	Z-FR-AMC	15.3 ± 4.1 nM
Bestatin		
Fr. No 26–27	R-AMC	247 ± 38 µM
Fr. No 35–36	Z-RR-AMC	No inhibition up to 1 mM
	Z-FR-AMC	No inhibition up to 1 mM
1,10-Phenanthroline		
Fr. No 26–27	R-AMC	68.5 ± 12.2 µM
Fr. No 35–36	Z-RR-AMC	No inhibition up to 5 mM
	Z-FR-AMC	No inhibition up to 5 mM

IC_50_ values are based on three technical replicates. For the inhibition analysis, fractions Nos. 26–27 and Nos. 35–36 were pooled and evaluated for cleavage of R-AMC or Z-RR-AMC/Z-FR-AMC substrates, respectively.

**Table 2:** The total number of peptide spectra identified as belonging to a specific PLCP and the proportion of amino acid sequence (% coverage) represented by the identified peptides detected by tandem MS analysis in two protein bands excised from SDS-PAGE.

Name	UniProt ID	Total Spectral Count (% of Coverage) *	
		43 kDa Band	38 kDa Band
*Hv*Pap-1	F2DDC9	5 (13)	47 (24)
*Hv*Pap-6	A0A8I6WYU4	270 (29)	257 (25)
*Hv*Pap-7	F2E6V2	8 (15)	6 (17)
*Hv*Pap-8	B4ESF0	5 (9.4)	13 (22)
*Hv*Pap-12	P05167	8 (14)	83 (26)
*Hv*Pap-13	A0A8I6XJP4	7 (16)	40 (44)
*Hv*Pap-14	B4ESF2	3 (9.5)	73 (37)
*Hv*Pap-16	B4ESF4	2 (4.9)	10 (21)
*Hv*Pap-17	A0A8I6Y6A5	6 (17)	12 (21)
*Hv*Pap-19	A0A8I7BBL8	7 (17)	40 (33)
*Hv*Pap-20	A0A8I7B6S7	5 (8.2)	17 (18)

* Data from one representative experiment.

**Table 3:** Classification of barley PLCPs, based on previously reported and the present phylogenetic tree analysis.

Name ^1^	Subfamily ^2^	Gene Identifier ^3^	UniProt ID	nAA ^4^	MW, Da ^5^
*Hv*Pap-1	F-like (RD19)	MOREX.r3.5HG0499760	F2DDC9	377	41,016
*Hv*Pap-2	F-like (RD19)	MOREX.r3.2HG0159420	A0A8I6WSN3	378	41,885
*Hv*Pap-3	F-like (RD19)	MOREX.r3.2HG0131510	B4ESE5	368	38,969
B4ESE4	F-like (RD19)	Akashinriki.Proj.2HG00143120	B4ESE4	381	41,931
F2E082	F-like (RD19)	N.F.	F2E082	341	37,057
*Hv*Pap-12	H-like (ALP)	MOREX.r3.5HG0480100	P05167	362	39,122
*Hv*Pap-19	B-like (CTB)	MOREX.r3.4HG0339740	A0A8I7BBL8	344	37,222
*Hv*Pap-20	B-like (CTB)	MOREX.r3.4HG0339730	A0A8I7B6S7	353	38,423
*Hv*Pap-30	B-like (CTB)	MOREX.r3.4HG0339750	A0A8I6X9J3	347	37,752
F2E819	B-like (CTB)	N.F.	F2E819	471	50,283
*Hv*Pap-17	L-like A (SAG12)	MOREX.r3.5HG0511140	A0A8I6Y6A5	349	36,895
A0A8I6YIB9	L-like A (SAG12)	MOREX.r3.6HG0570590	A0A8I6YIB9	343	36,737
A0A8I7B6Q5	L-like A (SAG12)	MOREX.r3.4HG0336140	A0A8I7B6Q5	341	36,789
A0A8I7B163	L-like A (SAG12)	MOREX.r3.1HG0061980	A0A8I7B163	339	36,752
A0A8I6Y7J0	L-like A (SAG12)	MOREX.r3.7HG0673530	A0A8I6Y7J0	340	36,792
A0A8I6Y5F2	L-like A (SAG12)	MOREX.r3.6HG0623260	A0A8I6Y5F2	339	37,020
A0A8I6X0V2	L-like A (SAG12)	MOREX.r3.2HG0103220	A0A8I6X0V2	340	37,030
A0A8I6WU74	L-like A (SAG12)	MOREX.r3.1HG0061970	A0A8I6WU74	340	37,333
*Hv*Pap-9	L-like B (CEP)	MOREX.r3.4HG0342040	A0A8I6XX70	365	40,033
*Hv*Pap-10	L-like B (CEP)	MOREX.r3.3HG0308010	A0A8I6XAH7	373	40,574
*Hv*Pap-14	L-like B (CEP)	MOREX.r3.3HG0304500	B4ESF2	367	39,790
*Hv*Pap-24	L-like B (CEP)	MOREX.r3.2HG0211390	A0A8I6WKU7	320	36,267
*Hv*Pap-40	L-like B (CEP)	MOREX.r3.6HG0548850	A0A8I6XXP5	374	41,375
*Hv*Pap-41	L-like B (CEP)	MOREX.r3.6HG0543660	A0A8I6XQ46	357	39,752
*Hv*Pap-42	L-like B (CEP)	MOREX.r3.3HG0308000	A0A8I7BAT3	379	40,979
A0A8I6YP35	L-like B (CEP)	MOREX.r3.6HG0545100	A0A8I6YP35	358	39,836
A0A8I6XQB9	L-like B (CEP)	MOREX.r3.6HG0545330	A0A8I6XQB9	231	24,863
A0A8I6XX42	L-like B (CEP)	MOREX.r3.6HG0545210	A0A8I6XX42	366	40,642
A0A8I6Y1D1	L-like B (CEP)	MOREX.r3.6HG0545130	A0A8I6Y1D1	338	37,546
A0A8I7BFP8	L-like B (CEP)	MOREX.r3.7HG0747000	A0A8I7BFP8	352	38,442
*Hv*Pap-4	L-like C (XCP)	MOREX.r3.5HG0462580	B4ESE6	356	38,651
*Hv*Pap-5	L-like C (XCP)	MOREX.r3.1HG0004220	B4ESE7	351	38,212
*Hv*Pap-26	L-like C (XCP)	MOREX.r3.6HG0609400	A0A287UKW8	364	39,959
*Hv*Pap-6	L-like D (RD21)	MOREX.r3.2HG0204520	A0A8I6WYU4	463	50,226
*Hv*Pap-7	L-like D (RD21)	MOREX.r3.2HG0212170	F2E6V2	473	50,677
*Hv*Pap-23	L-like D (RD21)	N.F.	B4ESF8	190	21,595
F2CQM4	L-like D (RD21)	N.F.	F2CQM4	436	47,292
A0A8I6X8Z3	L-like D (RD21)	MOREX.r3.2HG0211940	A0A8I6X8Z3	469	50,348
F2DC72	L-like D (RD21)	N.F.	F2DC72	289	31,994
A0A8I6YCJ2	L-like D (RD21)	MOREX.r3.7HG0685270	A0A8I6YCJ2	477	51,115
*Hv*Pap-13	L-like E	MOREX.r3.5HG0506230	A0A8I6XJP4	366	39,825
*Hv*Pap-15	L-like E	MOREX.r3.6HG0545500	M0YYX8	406	44,371
*Hv*Pap-16	L-like E	MOREX.r3.7HG0749670	B4ESF4	389	42,091
*Hv*Pap-18	L-like E	Akashinriki.Proj.4HG00376280	F2CR43	365	39,599
*Hv*Pap-21	L-like E	MOREX.r3.2HG0205780	A0A8I6WU79	355	38,776
*Hv*Pap-22	L-like E	MOREX.r3.1HG0004940	A0A8I6WPU1	345	37,350
*Hv*Pap-25	L-like E	MOREX.r3.1HG0085150	A0A8I7B430	355	38,295
*Hv*Pap-27	L-like E	MOREX.r3.3HG0245120	A0A8I6WQD4	357	38,641
*Hv*Pap-28	L-like E	MOREX.r3.5HG0424540	A0A8I6XSH6	396	43,119
*Hv*Pap-29	L-like E	MOREX.r3.3HG0245250	A0A8I7B9F2	359	38,974
*Hv*Pap-33	L-like E	MOREX.r3.2HG0104490	A0A8I6W9L7	355	37,217
*Hv*Pap-34	L-like E	MOREX.r3.2HG0106320	F2EC73	360	39,066
*Hv*Pap-35	L-like E	MOREX.r3.2HG0212960	A0A8I6WXF8	349	37,351
*Hv*Pap-36	L-like E	MOREX.r3.6HG0539010	A0A8I6YPN0	383	41,925
*Hv*Pap-37	L-like E	MOREX.r3.4HG0418460	F2DVR5	389	42,527
*Hv*Pap-38	L-like E	MOREX.r3.3HG0230090	A0A8I6XUD2	390	42,342
A0A8I6XNC2	L-like E	MOREX.r3.3HG0271470	A0A8I6XNC2	350	37,378
F2CPT6	L-like E	10TJ18.Proj.3HG00173970	F2CPT6	225	23,480
*Hv*Pap-8	(XBCP3)	MOREX.r3.1HG0076400	B4ESF0	457	48,431
A0A8I6XRJ4	(THI1)	MOREX.r3.5HG0492390	A0A8I6XRJ4	418	44,588
*Hv*Pap-31	N.S.	N.F.	F2E606	329	35,447
*Hv*Pap-32	N.S.	N.F.	F2E3H6	365	39,351
*Hv*Pap-39	N.S.	N.F.	F2E5Q3	333	37,013
A0A8I6WZM8	N.S.	MOREX.r3.2HG0208910	A0A8I6WZM8	330	36,201

^1^ HvPap number as introduced by Díaz-Mendoza et al. [8], or UniProt identifier [51,52]. ^2^ Subfamily name based on the phylogenetic tree analysis of Díaz-Mendoza et al. [8], Richau et al. [13] and the present study. ^3^ Gene identifier is based on cultivar ‘Morex’ v3 gene IDs (beginning with HORVU.MOREX.r3) or pangenome gene IDs (see https://panbarlex.ipk-gatersleben.de/; [53]). ^4^ Number of amino acids (nAA) in the predicted full-length protein sequence. ^5^ Molecular weight of the full-length protein sequence. N.S., the subfamily was not specified. N.F., the gene identifier has not been found among ‘Morex’ v3 gene models, or in the pangenome database.

**Table 4:** Comparison of *Hv*Pap-6 orthologs by pairwise sequence alignment.

Name	Species	UniProt ID	nAA	M.W., Da	Comparison with *Hv*Pap-6	
					Identity (%)	Similarity (%)
*Hv*Pap-6	*Hordeum vulgare* subsp*. vulgare*	A0A8I6WYU4	463	50,226	-	-
*Hv*Pap-7	*Hordeum vulgare* subsp*. vulgare*	F2E6V2	473	50,677	56.2	66.3
Triticain α	*Triticum aestivum*	Q0WXG8	461	50,407	90.2	92.3
Mir3	*Zea mays*	O22500	480	51,787	71.5	77.8
RD21A	*Arabidopsis thaliana*	P43297	462	50,966	62.2	73.6

In the pairwise sequence alignments, identity refers to the percentage of identical amino acids at corresponding positions in two aligned sequences; similarity considers not only identical amino acids but also those with similar physicochemical properties.

**Table 5:** Distribution of PLCPs over subfamilies for *H. vulgare*, *A. thaliana*, and *Zea mays.*

No.	Subfamily of PLCPs		*A. thaliana* by [13]	*H. vulgare* (Present Study)	*Z. mays*	
	By [13]	By [8]			By [37]	By [13]
1	RD21	Cathepsin L-like D	9	7	12	13
2	CEP	Cathepsin L-like B	3	12	7	3
3	XCP	Cathepsin L-like C	2	3	3	2
4	XBCP3	N.S.	1	1	1	1
5	THI1	N.S.	1	1	10	8
6	SAG12	Cathepsin L-like A	6	8	11	5
7	RD19	Cathepsin F-like	4	4	5	2
8	ALP	Cathepsin H-like	2	1	2	5
9	CTB	Cathepsin B-like	3	4	2	1
	N.S.	Cathepsin L-like E	N.F.	18	N.F.	N.F.

The distribution is presented as the number of PLCPs by subfamilies for plant species with more than 20 sequenced PLCPs. N.S., not specified; N.F., not found. The numbering of subfamilies for the first nine clusters is given as in [13].

## Data Availability

Data are contained within the article and Supplementary Materials.

## Supplementary Materials

The following supporting information can be downloaded at: https://www.mdpi.com/article/10.3390/plants14203132/s1. Figure S1. PLCPs from barley leaves were enriched using DEAE-Sepharose ion-exchange and affinity chromatography of DCG-04-labeled barley proteases, then separated on 4–12% SDS-PAGE and stained with Coomassie Blue. To demonstrate the specificity of DCG-04 labeling, the cathepsin inhibitor CAA0225 (30 μM) was added 30 min prior to labeling to block the binding of DCG-04 to PLCPs. Bands with molecular weights around 43 and 38 kDa (indicated with dotted rectangles) were excised and analyzed by tandem MS. Tables S1–S11. Peptide sequences of barley PLCPs, identified by tandem MS analysis. The samples were prepared as described in Section 3.5 (Gel-Based Tandem MS Analysis). Modification (+57) indicates a carbamidomethylation; (+16) indicates an oxidation; “Delta” is “Actual” minus “Calculated” peptide mass (Da); m/z is the mass-to-charge ratio. Table S12. Distribution of barley L-like E cathepsin orthologs across plant species.
