# Supplementary material for: Activity-Based Profiling of Papain-like Cysteine Proteases During Late-Stage Leaf Senescence in Barley
Source: Plants (Basel). 2025 Oct 11;14(20):3132. doi: 10.3390/plants14203132 (PMC12567500; doi:10.3390/plants14203132)
Supplement: Supplementary file 1 [file plants-14-03132-s001.zip › plants-3755401-supplementary.pdf]

Supplementary Materials

for

Activity-based Profiling of Papain-like Cysteine Proteases During Late-Stage Leaf Senescence in Barley

Igor A. Schepetkin and Andreas M. Fischer

Department of Plant Sciences and Plant Pathology, Montana State University, Bozeman, MT 59717, USA

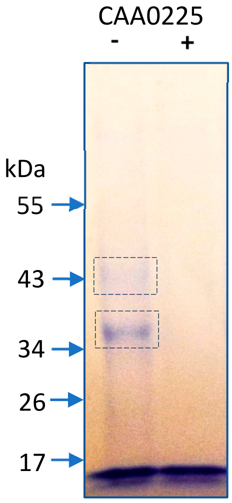

Figure S1. PLCPs from barley leaves were enriched using DEAE-Sepharose ion-exchange and affinity chromatography of DCG-04-labeled barley proteases, then separated on 4-12% SDS-PAGE and stained with Coomassie. To demonstrate the specificity of DCG-04 labeling, the cathepsin inhibitor CAA0225 (30 μM) was added 30 min prior to labeling to block the binding of DCG-04 to PLCPs. Bands with molecular weights around 43 and 38 kDa (indicated with dotted rectangles) were excised and analyzed by tandem MS.

Supplementary Tables S1–S11. Peptide sequences of barley PLCP ionized by electrospray followed by tandem MS analysis. The samples were prepared as described in section *3.5* (*Gel-Based Tandem MS Analysis*). Modification (+57) indicates a carbamidomethylation; (+16) indicates an oxidation; "Delta" is "Actual" minus "Calculated" peptide mass (Da); m/z is the mass-to-charge ratio.

Table S1. Peptide sequences of *Hv*Pap-1 (F2DDC9) with 11 exclusive unique peptides, 20 exclusive unique spectra, 47 total spectra, and 24% coverage (89/377 amino acids). The sample (representative of three experiments) was excised from the 38-kDa band of SDS-PAGE.

Peptide Sequence	Probability	Ion Score	Modifications	Observed m/z	Charge	Delta Da
(R)DHGAVGPVK(N)	100%	42.9		440.2377	2	-0.0003799
(R)DHGAVGPVK(N)	100%	31.22		440.2379	2	-0.00001394
(R)DHGAVGPVK(N)	100%	30.46		440.2378	2	-0.0001979
(R)EKDYPYTGK(D)	99%	29.69		550.7662	2	-0.0008519
(K)DYPYTGK(D)	27%	10.26		422.1978	2	-0.0001539
(K)DYPYTGK(D)	7%	5.88		422.1978	2	-0.0002139
(K)DGTcKFDK(S)	99%	29.77	(+57)	485.7184	2	-0.0005592
(R)HLDHGVLLVGYGASGFAPSR(F)	100%	73.9		685.0253	3	-0.0001079
(R)HLDHGVLLVGYGASGFAPSR(F)	100%	73.01		685.0254	3	0.00007506
(R)HLDHGVLLVGYGASGFAPSR(F)	100%	70.7		685.0256	3	0.0006241
(R)HLDHGVLLVGYGASGFAPSR(F)	100%	70.56		514.0207	4	-0.0006099
(R)HLDHGVLLVGYGASGFAPSR(F)	100%	69.46		685.0252	3	-0.0004739
(R)HLDHGVLLVGYGASGFAPSR(F)	100%	69.39		514.0208	4	-0.0001219
(R)HLDHGVLLVGYGASGFAPSR(F)	100%	69.35		514.0209	4	0.0001221
(R)HLDHGVLLVGYGASGFAPSR(F)	100%	67.4		685.0256	3	0.0008071
(R)HLDHGVLLVGYGASGFAPSR(F)	100%	65.35		514.0211	4	0.0008541
(R)HLDHGVLLVGYGASGFAPSR(F)	100%	62.79		514.0209	4	0.0003661
(R)HLDHGVLLVGYGASGFAPSR(F)	100%	54.84		514.0209	4	0.0003661
(R)HLDHGVLLVGYGASGFAPSR(F)	100%	54.52		514.0211	4	0.0008541
(R)HLDHGVLLVGYGASGFAPSR(F)	100%	51.54		514.0209	4	0.0001221
(R)HLDHGVLLVGYGASGFAPSR(F)	100%	48.78		685.0255	3	0.0002581
(R)HLDHGVLLVGYGASGFAPSR(F)	100%	34.59		685.0255	3	0.0002581
(R)HLDHGVLLVGYGASGFAPSR(F)	100%	32.72		685.0256	3	0.0006241
(R)HLDHGVLLVGYGASGFAPSR(F)	100%	26.58		514.0208	4	-0.0001219
(R)HLDHGVLLVGYGASGFAPSR(F)	100%	36.67		1,027.04	2	0.001246
(R)HLDHGVLLVGYGASGFAPSR(F)	99%	20.36		685.0253	3	-0.0001079
(R)FKEKPYWIIK(N)	100%	31.07		451.264	3	-0.00006694
(R)FKEKPYWIIK(N)	100%	24.69		451.2638	3	-0.0006159
(R)FKEKPYWIIK(N)	99%	20.7		451.2639	3	-0.0004329
(R)FKEKPYWIIK(N)	35%	11.05		676.3923	2	-0.0001759

(K)EKPYWIIK(N)	99%	31.78		538.8105	2	-0.0002279
(K)EKPYWIIK(N)	15%	8.36		538.8105	2	-0.0003499
(K)NSWGENWGDK(G)	100%	37.39		596.7547	2	0.0002781
(K)NSWGENWGDK(G)	11%	7.43		596.7545	2	-0.0002099
(K)NSWGENWGDKGYYK(I)	100%	52.8		852.3756	2	-0.001002
(K)NSWGENWGDKGYYK(I)	100%	28.17		568.5865	3	-0.00003694
(K)NSWGENWGDKGYYK(I)	100%	27.45		568.5862	3	-0.0007689
(K)cGVDSmVSTVSATHSSK(E)	100%	53.69	(+57), (+16)	884.8947	2	-0.001874
(K)cGVDSmVSTVSATHSSK(E)	100%	29.8	(+57), (+16)	590.266	3	-0.0006053
(K)cGVDSMVSTVSATHSSK(E)	100%	28.65	(+57), (+16)	584.9343	3	-0.0006852
(K)cGVDSmVSTVSATHSSK(E)	100%	23.01	(+57), (+16)	590.2665	3	0.001045
(K)cGVDSmVSTVSATHSSKEE(-)	100%	79.79	(+57), (+16)	676.2943	3	-0.0008423
(K)cGVDSMVSTVSATHSSKEE(-)	100%	62.78	(+57)	670.9636	3	0.002006
(K)cGVDSmVSTVSATHSSKEE(-)	100%	58.08	(+57), (+16)	1,013.94	2	-0.0008923
(K)cGVDSmVSTVSATHSSKEE(-)	100%	40.69	(+57), (+16)	676.2948	3	0.0006217
(K)cGVDSMVSTVSATHSSKEE(-)	97%	19.49	(+57)	670.9635	3	0.00164
(K)cGVDSMVSTVSATHSSKEE(-)	95%	26.6	(+57)	1,005.94	2	-0.002559

Table S2. Peptide sequences of *Hv*Pap-6 (A0A8I6WYU4) with 16 exclusive unique peptides, 25 exclusive unique spectra, 270 total spectra, and 29% coverage (141/492 amino acids). The sample (representative of three experiments) was excised from the 43-kDa band of SDS-PAGE.

Peptide Sequence	Probability	Ion Score	Modifi-cations	Observed m/z	Charge	Delta Da
(R)STYLGAR(T)	19%	9.25		384.206	2	0.00006606
(R)YQAADNDELPESVDWR(K)	100%	138.43		954.4248	2	0.001588
(R)YQAADNDELPESVDWR(K)	100%	32.69		636.619	3	0.001576
(R)YQAADNDELPESVDWR(K)	100%	34.84		954.4235	2	-0.0009759
(R)YQAADNDELPESVDWR(K)	87%	18.66		954.4238	2	-0.0003659
(R)YQAADNDELPESVDWRK(K)	100%	29.48		679.3165	3	-0.0007139
(R)KKGAVGAVK(D)	84%	17.65		429.2819	2	-0.0003399
(K)KNAKVVTIDGYEDVPVNSEK(S)	96%	17.79		735.7177	3	-0.001545
(K)NAKVVTIDGYEDVPVNSEK(S)	100%	76.63		693.0199	3	0.0001931
(K)NAKVVTIDGYEDVPVNSEK(S)	100%	54.08		1,039.03	2	-0.0002199
(K)NAKVVTIDGYEDVPVNSEK(S)	97%	17.07		693.0195	3	-0.0009049
(K)VVTIDGYEDVPVNSEK(S)	100%	121.75		882.4379	2	-0.00149
(K)VVTIDGYEDVPVNSEK(S)	100%	113.17		882.4383	2	-0.0006359
(K)VVTIDGYEDVPVNSEK(S)	100%	108.39		882.4384	2	-0.0003899
(K)VVTIDGYEDVPVNSEK(S)	100%	108.25		882.4385	2	-0.0002679
(K)VVTIDGYEDVPVNSEK(S)	100%	107.14		882.4379	2	-0.001368
(K)VVTIDGYEDVPVNSEK(S)	100%	103.75		882.4382	2	-0.0007579
(K)VVTIDGYEDVPVNSEK(S)	100%	103.22		882.4382	2	-0.0008799

(K)VVTIDGYEDVPVNSEK(S)	100%	97.32		882.4376	2	-0.001978
(K)VVTIDGYEDVPVNSEK(S)	100%	96.58		882.4377	2	-0.001734
(K)VVTIDGYEDVPVNSEK(S)	100%	96.03		882.438	2	-0.001124
(K)VVTIDGYEDVPVNSEK(S)	100%	95.83		882.4383	2	-0.0006359
(K)VVTIDGYEDVPVNSEK(S)	100%	95.16		882.4386	2	-0.00002394
(K)VVTIDGYEDVPVNSEK(S)	100%	95.12		882.4386	2	-0.00002394
(K)VVTIDGYEDVPVNSEK(S)	100%	94.78		882.4379	2	-0.001368
(K)VVTIDGYEDVPVNSEK(S)	100%	94.14		882.4381	2	-0.001002
(K)VVTIDGYEDVPVNSEK(S)	100%	93.75		882.438	2	-0.001124
(K)VVTIDGYEDVPVNSEK(S)	100%	93.53		882.4385	2	-0.0002679
(K)VVTIDGYEDVPVNSEK(S)	100%	90.34		882.4381	2	-0.001002
(K)VVTIDGYEDVPVNSEK(S)	100%	90.13		882.4384	2	-0.0005139
(K)VVTIDGYEDVPVNSEK(S)	100%	89.97		882.4383	2	-0.0006359
(K)VVTIDGYEDVPVNSEK(S)	100%	89.61		882.4382	2	-0.0008799
(K)VVTIDGYEDVPVNSEK(S)	100%	86.08		882.438	2	-0.001246
(K)VVTIDGYEDVPVNSEK(S)	100%	85.2		882.4377	2	-0.001734
(K)VVTIDGYEDVPVNSEK(S)	100%	84.01		882.4384	2	-0.0003899
(K)VVTIDGYEDVPVNSEK(S)	100%	80.57		882.4387	2	0.0002201
(K)VVTIDGYEDVPVNSEK(S)	100%	79.36		882.4383	2	-0.0006359
(K)VVTIDGYEDVPVNSEK(S)	100%	78.41		882.4372	2	-0.002832
(K)VVTIDGYEDVPVNSEK(S)	100%	77.51		882.439	2	0.0008301
(K)VVTIDGYEDVPVNSEK(S)	100%	77.45		882.4384	2	-0.0005139
(K)VVTIDGYEDVPVNSEK(S)	100%	77.29		882.4382	2	-0.0008799
(K)VVTIDGYEDVPVNSEK(S)	100%	77.15		882.4385	2	-0.0002679
(K)VVTIDGYEDVPVNSEK(S)	100%	77.07		882.4382	2	-0.0008799
(K)VVTIDGYEDVPVNSEK(S)	100%	76.83		882.4386	2	-0.00002394
(K)VVTIDGYEDVPVNSEK(S)	100%	76.75		882.4382	2	-0.0008799
(K)VVTIDGYEDVPVNSEK(S)	100%	76.72		882.4382	2	-0.0008799
(K)VVTIDGYEDVPVNSEK(S)	100%	76.33		882.438	2	-0.001124
(K)VVTIDGYEDVPVNSEK(S)	100%	74.45		882.4389	2	0.0005861
(K)VVTIDGYEDVPVNSEK(S)	100%	73.79		882.4387	2	0.00009806
(K)VVTIDGYEDVPVNSEK(S)	100%	61.05		588.6281	3	-0.0003419
(K)VVTIDGYEDVPVNSEK(S)	100%	59.9		588.6281	3	-0.0001589
(K)VVTIDGYEDVPVNSEK(S)	100%	59.14		588.6279	3	-0.0007079
(K)VVTIDGYEDVPVNSEK(S)	100%	73.5		882.4388	2	0.0003421
(K)VVTIDGYEDVPVNSEK(S)	100%	73.33		882.4392	2	0.001196
(K)VVTIDGYEDVPVNSEK(S)	100%	72.47		882.4385	2	-0.0001459
(K)VVTIDGYEDVPVNSEK(S)	100%	71.54		882.4384	2	-0.0005139
(K)VVTIDGYEDVPVNSEK(S)	100%	71.5		882.4382	2	-0.0008799
(K)VVTIDGYEDVPVNSEK(S)	100%	71.49		882.4383	2	-0.0006359
(K)VVTIDGYEDVPVNSEK(S)	100%	69.89		882.4377	2	-0.001856

(K)VVTIDGYEDVPVNSEK(S)	100%	68.11		882.4379	2	-0.001368
(K)VVTIDGYEDVPVNSEK(S)	100%	67.91		882.438	2	-0.001246
(K)VVTIDGYEDVPVNSEK(S)	100%	65.66		882.4383	2	-0.0006359
(K)VVTIDGYEDVPVNSEK(S)	100%	64.26		882.4384	2	-0.0003899
(K)VVTIDGYEDVPVNSEK(S)	100%	64.99		882.4388	2	0.0004641
(K)VVTIDGYEDVPVNSEK(S)	100%	67.12		882.4393	2	0.001318
(K)VVTIDGYEDVPVNSEK(S)	100%	63.56		882.4382	2	-0.0007579
(K)VVTIDGYEDVPVNSEK(S)	100%	63.13		882.4383	2	-0.0006359
(K)VVTIDGYEDVPVNSEK(S)	100%	60.36		882.4384	2	-0.0005139
(K)VVTIDGYEDVPVNSEK(S)	100%	60.33		882.4384	2	-0.0005139
(K)VVTIDGYEDVPVNSEK(S)	100%	60.65		882.438	2	-0.001124
(K)VVTIDGYEDVPVNSEK(S)	100%	60.5		882.4388	2	0.0004641
(K)VVTIDGYEDVPVNSEK(S)	100%	60.94		882.4377	2	-0.001856
(K)VVTIDGYEDVPVNSEK(S)	100%	40.28		588.6279	3	-0.0007079
(K)VVTIDGYEDVPVNSEK(S)	100%	57.85		882.4384	2	-0.0003899
(K)VVTIDGYEDVPVNSEK(S)	100%	58.93		882.4379	2	-0.00149
(K)VVTIDGYEDVPVNSEK(S)	100%	57.18		882.4389	2	0.0005861
(K)VVTIDGYEDVPVNSEK(S)	100%	54.36		882.4385	2	-0.0002679
(K)VVTIDGYEDVPVNSEK(S)	100%	57.92		882.4393	2	0.001318
(K)VVTIDGYEDVPVNSEK(S)	100%	53.52		882.4385	2	-0.0001459
(K)VVTIDGYEDVPVNSEK(S)	100%	52.71		882.4376	2	-0.001978
(K)VVTIDGYEDVPVNSEK(S)	100%	48.76		882.4378	2	-0.001612
(K)VVTIDGYEDVPVNSEK(S)	100%	45.93		882.4388	2	0.0003421
(K)VVTIDGYEDVPVNSEK(S)	100%	46.93		882.4374	2	-0.002344
(K)VVTIDGYEDVPVNSEK(S)	100%	26.25		588.628	3	-0.0005249
(K)VVTIDGYEDVPVNSEK(S)	100%	38.48		882.4383	2	-0.0006359
(K)VVTIDGYEDVPVNSEK(S)	100%	41.05		882.4392	2	0.001196
(K)VVTIDGYEDVPVNSEK(S)	100%	36.24		882.4389	2	0.0005861
(K)VVTIDGYEDVPVNSEK(S)	100%	24.4		588.6286	3	0.001305
(K)VVTIDGYEDVPVNSEK(S)	100%	22.17		588.6284	3	0.0007561
(K)VVTIDGYEDVPVNSEK(S)	99%	18.74		588.6281	3	-0.0003419
(K)VVTIDGYEDVPVNSEK(S)	99%	25.32		588.6274	3	-0.002175
(K)VVTIDGYEDVPVNSEK(S)	98%	25.1		882.4387	2	0.0002201
(K)VVTIDGYEDVPVNSEK(S)	96%	22.47		882.4385	2	-0.0002679
(K)VVTIDGYEDVPVNSEK(S)	85%	15.17		588.6277	3	-0.00144
(K)VVTIDGYEDVPVNSEK(S)	67%	9.75		588.6281	3	-0.0001589
(K)VVTIDGYEDVPVNSEK(S)	54%	13.53		882.4386	2	-0.00002394
(K)VVTIDGYEDVPVNSEK(S)	34%	9.72		588.6287	3	0.001488
(K)VVTIDGYEDVPVNSEK(S)	13%	8.63		882.438	2	-0.001246
(K)VVTIDGYEDVPVNSEK(S)	8%	4.98		588.6279	3	-0.0008909
(K)VVTIDGYEDVPVNSEKSLQK(A)	100%	53.71		740.718	3	-0.0001439

(K)VVTIDGYEDVPVNSEKSLQK(A)	100%	49.23		740.7182	3	0.0004051
(K)VVTIDGYEDVPVNSEKSLQK(A)	100%	34.21		740.7172	3	-0.002526
(K)VVTIDGYEDVPVNSEKSLQK(A)	75%	11.06		740.7178	3	-0.0006959
(K)VVTIDGYEDVPVNSEKSLQK(A)	69%	10.26		740.7182	3	0.0004051
(K)SLQKAVANQPISVAIEAGGR(A)	100%	81.41		1,005.06	2	0.0004821
(K)SLQKAVANQPISVAIEAGGR(A)	100%	58.8		670.3763	3	0.0005311
(K)SLQKAVANQPISVAIEAGGR(A)	99%	20.55		670.3765	3	0.0008971
(K)SLQKAVANQPISVAIEAGGR(A)	52%	14.67		670.3753	3	-0.002583
(K)AVANQPISVAIEAGGR(A)	100%	83.78		776.9257	2	-0.00008194
(K)AVANQPISVAIEAGGR(A)	100%	82.29		776.9257	2	-0.0002039
(K)AVANQPISVAIEAGGR(A)	100%	82.21		776.9255	2	-0.0004479
(K)AVANQPISVAIEAGGR(A)	100%	79.82		776.9254	2	-0.0006919
(K)AVANQPISVAIEAGGR(A)	100%	75.75		776.9255	2	-0.0005699
(K)AVANQPISVAIEAGGR(A)	100%	71.3		518.2861	3	-0.0003989
(K)AVANQPISVAIEAGGR(A)	100%	63.61		518.2862	3	-0.0002159
(K)AVANQPISVAIEAGGR(A)	100%	55.29		518.2868	3	0.001614
(K)AVANQPISVAIEAGGR(A)	100%	75.83		776.9264	2	0.001262
(K)AVANQPISVAIEAGGR(A)	100%	73.49		776.9256	2	-0.0003259
(K)AVANQPISVAIEAGGR(A)	100%	73		776.9256	2	-0.0003259
(K)AVANQPISVAIEAGGR(A)	100%	70.3		776.9256	2	-0.0003259
(K)AVANQPISVAIEAGGR(A)	100%	70.19		776.9259	2	0.0002841
(K)AVANQPISVAIEAGGR(A)	100%	69.47		776.9256	2	-0.0003259
(K)AVANQPISVAIEAGGR(A)	100%	68.65		776.9259	2	0.0002841
(K)AVANQPISVAIEAGGR(A)	100%	67.94		776.9257	2	-0.0002039
(K)AVANQPISVAIEAGGR(A)	100%	68.0		776.9258	2	0.0001621
(K)AVANQPISVAIEAGGR(A)	100%	67.99		776.9253	2	-0.0009359
(K)AVANQPISVAIEAGGR(A)	100%	66.61		776.9255	2	-0.0005699
(K)AVANQPISVAIEAGGR(A)	100%	66.53		776.9255	2	-0.0004479
(K)AVANQPISVAIEAGGR(A)	100%	43.71		518.2863	3	-0.00003294
(K)AVANQPISVAIEAGGR(A)	100%	65.97		776.9252	2	-0.001058
(K)AVANQPISVAIEAGGR(A)	100%	65.93		776.926	2	0.0005281
(K)AVANQPISVAIEAGGR(A)	100%	64.91		776.925	2	-0.001424
(K)AVANQPISVAIEAGGR(A)	100%	62.0		776.9255	2	-0.0005699
(K)AVANQPISVAIEAGGR(A)	100%	59.33		776.926	2	0.0004061
(K)AVANQPISVAIEAGGR(A)	100%	37.66		518.2863	3	-0.00003294
(K)AVANQPISVAIEAGGR(A)	100%	36.47		518.2862	3	-0.0002159
(K)AVANQPISVAIEAGGR(A)	100%	63.35		776.9246	2	-0.0024
(K)AVANQPISVAIEAGGR(A)	100%	54.27		776.9255	2	-0.0004479
(K)AVANQPISVAIEAGGR(A)	100%	53.95		776.9252	2	-0.00118
(K)AVANQPISVAIEAGGR(A)	100%	50.06		776.9251	2	-0.001302
(K)AVANQPISVAIEAGGR(A)	100%	35.04		518.2859	3	-0.001131

(K)AVANQPISVAIEAGGR(A)	100%	48.15		776.9259	2	0.0002841
(K)AVANQPISVAIEAGGR(A)	100%	48.91		776.9261	2	0.0006501
(K)AVANQPISVAIEAGGR(A)	100%	45.37		776.9254	2	-0.0008139
(K)AVANQPISVAIEAGGR(A)	100%	44.04		776.9257	2	-0.00008194
(K)AVANQPISVAIEAGGR(A)	100%	45.13		776.925	2	-0.001424
(K)AVANQPISVAIEAGGR(A)	100%	42.83		776.9257	2	-0.0002039
(K)AVANQPISVAIEAGGR(A)	100%	42.53		776.9255	2	-0.0004479
(K)AVANQPISVAIEAGGR(A)	100%	42.52		776.9254	2	-0.0008139
(K)AVANQPISVAIEAGGR(A)	100%	45.23		776.9263	2	0.001018
(K)AVANQPISVAIEAGGR(A)	100%	41.11		776.9256	2	-0.0003259
(K)AVANQPISVAIEAGGR(A)	100%	41.09		776.9256	2	-0.0003259
(K)AVANQPISVAIEAGGR(A)	100%	42.5		776.9251	2	-0.001302
(K)AVANQPISVAIEAGGR(A)	100%	40.62		776.9253	2	-0.0009359
(K)AVANQPISVAIEAGGR(A)	100%	40.03		776.9255	2	-0.0005699
(K)AVANQPISVAIEAGGR(A)	100%	41.03		776.926	2	0.0004061
(K)AVANQPISVAIEAGGR(A)	100%	39.51		776.9254	2	-0.0006919
(K)AVANQPISVAIEAGGR(A)	100%	37.96		776.9257	2	-0.0002039
(K)AVANQPISVAIEAGGR(A)	100%	37.33		776.9257	2	-0.0002039
(K)AVANQPISVAIEAGGR(A)	100%	36.74		776.9256	2	-0.0003259
(K)AVANQPISVAIEAGGR(A)	100%	35.74		776.9252	2	-0.00118
(K)AVANQPISVAIEAGGR(A)	100%	34.14		776.9257	2	-0.00008194
(K)AVANQPISVAIEAGGR(A)	100%	33.97		776.9254	2	-0.0008139
(K)AVANQPISVAIEAGGR(A)	100%	33.21		776.9257	2	-0.00008194
(K)AVANQPISVAIEAGGR(A)	100%	32.77		776.9254	2	-0.0008139
(K)AVANQPISVAIEAGGR(A)	100%	34.16		776.925	2	-0.001546
(K)AVANQPISVAIEAGGR(A)	100%	31.37		776.9255	2	-0.0004479
(K)AVANQPISVAIEAGGR(A)	100%	20.95		518.2861	3	-0.0005819
(K)AVANQPISVAIEAGGR(A)	100%	33.21		776.9263	2	0.001018
(K)AVANQPISVAIEAGGR(A)	100%	29.82		776.9255	2	-0.0004479
(K)AVANQPISVAIEAGGR(A)	100%	29.53		776.9255	2	-0.0004479
(K)AVANQPISVAIEAGGR(A)	99%	32.26		776.9266	2	0.001628
(K)AVANQPISVAIEAGGR(A)	99%	27.05		776.9255	2	-0.0004479
(K)AVANQPISVAIEAGGR(A)	99%	26.79		776.9255	2	-0.0005699
(K)AVANQPISVAIEAGGR(A)	96%	28.69		776.9268	2	0.002116
(K)AVANQPISVAIEAGGR(A)	95%	22.03		776.9257	2	-0.00008194
(K)AVANQPISVAIEAGGR(A)	95%	21.62		776.9254	2	-0.0006919
(K)AVANQPISVAIEAGGR(A)	93%	21.08		776.9258	2	0.0001621
(K)AVANQPISVAIEAGGR(A)	91%	20.00		776.9258	2	0.00004006
(K)AVANQPISVAIEAGGR(A)	86%	20.45		776.925	2	-0.001546
(K)AVANQPISVAIEAGGR(A)	85%	18.22		776.9258	2	0.00004006
(K)AVANQPISVAIEAGGR(A)	84%	19.09		776.9251	2	-0.001302

(K)AVANQPISVAIEAGGR(A)	84%	18.08		776.9253	2	-0.0009359
(K)AVANQPISVAIEAGGR(A)	83%	18.32		776.9252	2	-0.001058
(K)AVANQPISVAIEAGGR(A)	81%	17.87		776.9252	2	-0.001058
(K)AVANQPISVAIEAGGR(A)	76%	20.08		776.9248	2	-0.001912
(K)AVANQPISVAIEAGGR(A)	75%	21.25		776.9247	2	-0.002156
(K)AVANQPISVAIEAGGR(A)	65%	19.22		776.9266	2	0.00175
(K)AVANQPISVAIEAGGR(A)	64%	14.72		776.9257	2	-0.00008194
(K)AVANQPISVAIEAGGR(A)	56%	13.87		776.9258	2	0.00004006
(K)AVANQPISVAIEAGGR(A)	42%	12.26		776.9254	2	-0.0008139
(K)AVANQPISVAIEAGGR(A)	40%	12.36		776.9258	2	0.0001621
(K)AVANQPISVAIEAGGR(A)	39%	11.94		776.9257	2	-0.0002039
(K)AVANQPISVAIEAGGR(A)	23%	10.17		776.9258	2	0.0001621
(K)AVANQPISVAIEAGGR(A)	16%	7.76		518.2859	3	-0.001131
(K)AVANQPISVAIEAGGR(A)	9%	4.36		518.2864	3	0.0003331
(K)SGIFTGTcGTALDHGVAAVGYGTENGK(D)	100%	49.99	(+57)	880.75	3	-0.0007632
(K)SGIFTGTcGTALDHGVAAVGYGTENGK(D)	99%	17.3	(+57)	880.7501	3	-0.0005802
(K)SGIFTGTcGTALDHGVAAVGYGTENGK(D)	93%	14.26	(+57)	660.8143	4	-0.0009592
(R)NSWGSVWGEDGYIR(M)	100%	80.95		813.3718	2	0.001908
(R)NSWGSVWGEDGYIR(M)	100%	80.11		813.3706	2	-0.0004119
(R)NSWGSVWGEDGYIR(M)	100%	74.82		813.3705	2	-0.0005339
(R)NSWGSVWGEDGYIR(M)	100%	73.54		813.3704	2	-0.0007779
(R)NSWGSVWGEDGYIR(M)	100%	72.34		813.3704	2	-0.0007779
(R)NSWGSVWGEDGYIR(M)	100%	70.93		813.3704	2	-0.0008999
(R)NSWGSVWGEDGYIR(M)	100%	70.26		813.3709	2	0.00007806
(R)NSWGSVWGEDGYIR(M)	100%	68.96		813.3706	2	-0.0004119
(R)NSWGSVWGEDGYIR(M)	100%	67.76		813.3709	2	0.0002001
(R)NSWGSVWGEDGYIR(M)	100%	67.13		813.3705	2	-0.0005339
(R)NSWGSVWGEDGYIR(M)	100%	64.71		813.3707	2	-0.0002879
(R)NSWGSVWGEDGYIR(M)	100%	62.61		813.3705	2	-0.0006559
(R)NSWGSVWGEDGYIR(M)	100%	60.29		813.3707	2	-0.0002879
(R)NSWGSVWGEDGYIR(M)	100%	59.18		813.3706	2	-0.0004119
(R)NSWGSVWGEDGYIR(M)	100%	58.57		813.3704	2	-0.0007779
(R)NSWGSVWGEDGYIR(M)	100%	58.8		813.3703	2	-0.001022
(R)NSWGSVWGEDGYIR(M)	100%	57.69		813.3707	2	-0.0002879
(R)NSWGSVWGEDGYIR(M)	100%	58.15		813.3709	2	0.0002001
(R)NSWGSVWGEDGYIR(M)	100%	57.56		813.3708	2	-0.00004394
(R)NSWGSVWGEDGYIR(M)	100%	58.87		813.3701	2	-0.001388
(R)NSWGSVWGEDGYIR(M)	100%	55.89		813.3707	2	-0.0002879
(R)NSWGSVWGEDGYIR(M)	100%	55.24		813.3707	2	-0.0001659
(R)NSWGSVWGEDGYIR(M)	100%	55.65		813.3709	2	0.0002001
(R)NSWGSVWGEDGYIR(M)	100%	58.23		813.3713	2	0.001054

(R)NSWGSVWGEDGYIR(M)	100%	53.79		813.371	2	0.0003221
(R)NSWGSVWGEDGYIR(M)	100%	52.54		813.3709	2	0.00007806
(R)NSWGSVWGEDGYIR(M)	100%	52.11		813.3706	2	-0.0004119
(R)NSWGSVWGEDGYIR(M)	100%	52.64		813.3703	2	-0.001022
(R)NSWGSVWGEDGYIR(M)	100%	52.12		813.3708	2	-0.00004394
(R)NSWGSVWGEDGYIR(M)	100%	52.12		813.3708	2	-0.00004394
(R)NSWGSVWGEDGYIR(M)	100%	34.09		542.5831	3	0.0003091
(R)NSWGSVWGEDGYIR(M)	100%	54.13		813.3715	2	0.00142
(R)NSWGSVWGEDGYIR(M)	100%	28.84		542.5829	3	-0.00005694
(R)NSWGSVWGEDGYIR(M)	100%	44.59		813.3699	2	-0.001754
(R)NSWGSVWGEDGYIR(M)	100%	41.12		813.3706	2	-0.0004119
(R)NSWGSVWGEDGYIR(M)	100%	39.82		813.3707	2	-0.0002879
(R)NSWGSVWGEDGYIR(M)	100%	31.22		813.3707	2	-0.0001659
(R)NSWGSVWGEDGYIR(M)	100%	29.63		813.3705	2	-0.0005339
(R)NSWGSVWGEDGYIR(M)	99%	28.05		813.3706	2	-0.0004119
(R)NSWGSVWGEDGYIR(M)	66%	9.64		542.5829	3	-0.00005694
(R)NSWGSVWGEDGYIRmER(N)	96%	14.85	(+16)	686.6425	3	-0.000462
(R)NSWGSVWGEDGYIRmER(N)	88%	16.65	(+16)	686.6434	3	0.002286
(K)ASSGKcGIAVEPSYPTK(T)	100%	36.94	(+57)	584.6259	3	-0.0004012
(K)ASSGKcGIAVEPSYPTK(T)	98%	26.71	(+57)	876.4357	2	0.0006508
(K)ASSGKcGIAVEPSYPTK(T)	6%	5.41	(+57)	584.6264	3	0.001249
(K)cGIAVEPSYPTK(T)	100%	76.56	(+57)	661.3266	2	0.0001108
(K)cGIAVEPSYPTK(T)	100%	74.74	(+57)	661.3265	2	-0.00001124
(K)cGIAVEPSYPTK(T)	100%	67.74	(+57)	661.3265	2	-0.00001124
(K)cGIAVEPSYPTK(T)	100%	67.79	(+57)	661.3262	2	-0.0007432
(K)cGIAVEPSYPTK(T)	100%	65.48	(+57)	661.3264	2	-0.0002552
(K)cGIAVEPSYPTK(T)	100%	68.2	(+57)	661.3273	2	0.001455
(K)cGIAVEPSYPTK(T)	100%	52.77	(+57)	661.3262	2	-0.0006212
(K)cGIAVEPSYPTK(T)	100%	53.41	(+57)	661.3267	2	0.0003548
(K)cGIAVEPSYPTK(T)	100%	51.97	(+57)	661.3265	2	-0.00001124
(K)cGIAVEPSYPTK(T)	100%	51	(+57)	661.3263	2	-0.0004992
(K)cGIAVEPSYPTK(T)	100%	50.54	(+57)	661.3263	2	-0.0004992
(K)cGIAVEPSYPTK(T)	100%	47.37	(+57)	661.3264	2	-0.0003772
(K)cGIAVEPSYPTK(T)	100%	51.28	(+57)	661.3257	2	-0.001597
(K)cGIAVEPSYPTK(T)	100%	28.63	(+57)	441.2205	3	0.001107
(K)cGIAVEPSYPTK(T)	100%	36.5	(+57)	661.3265	2	-0.0001332
(K)cGIAVEPSYPTK(T)	100%	35.99	(+57)	661.3265	2	-0.0001332
(K)cGIAVEPSYPTK(T)	100%	37.68	(+57)	661.3268	2	0.0005988
(K)cGIAVEPSYPTK(T)	100%	34.72	(+57)	661.3264	2	-0.0003772
(K)cGIAVEPSYPTK(T)	99%	28.25	(+57)	661.3267	2	0.0002328
(K)cGIAVEPSYPTK(T)	99%	32.67	(+57)	661.3257	2	-0.001719

(K)cGIAVEPSYPTK(T)	98%	24.13	(+57)	661.3265	2	-0.00001124
(K)cGIAVEPSYPTK(T)	67%	15.73	(+57)	661.3261	2	-0.0008652
(K)cGIAVEPSYPTK(T)	61%	15.1	(+57)	661.3267	2	0.0003548
(K)cGIAVEPSYPTK(T)	21%	9.47	(+57)	661.3264	2	-0.0003772
(K)cGIAVEPSYPTK(T)	12%	12.8	(+57)	661.3275	2	0.001821
(K)QGTcLAAK(D)	99%	34.61	(+57)	424.7183	2	-0.0003032

Table S3. Peptide sequences of *Hv*Pap-7 (F2E6V2) with 6 exclusive unique peptides, 6 exclusive unique spectra, 6 total spectra, and 17% coverage (80/473 amino acids). The sample (representative of three experiments) was excised from the 38-kDa band of SDS-PAGE.

Peptide Sequence	Probability	Ion Score	Observed m/z	Charge	Delta Da
(K)NGGIDTEDDYPYK(A)	100%	73.55	743.8203	2	-0.0003199
(K)VVSIDGFEDVPENDEK(S)	13%	16.31	896.4161	2	-0.003668
(K)AVAHHPVSVAIEAGGR(E)	8%	8.89	393.4659	4	-0.003026
(R)EFQLYHSGVFSGR(C)	100%	29.2	509.5845	3	0.0002211
(R)NSWGPNWGEAGYLR(M)	7%	6.94	803.8729	2	-0.001294
(R)NINVTSGK(C)	7%	6.11	416.7297	2	-0.0001499

Table S4. Peptide sequences of *Hv*Pap-8 (B4ESF0) with 8 exclusive unique peptides, 11 exclusive unique spectra, 13 total spectra, and 22% coverage (100/457 amino acids). The sample (representative of three experiments) was excised from the 38-kDa band of SDS-PAGE.

Peptide Sequence	Probability	Ion Score	Modifications	Observed m/z	Charge	Delta Da
(K)ITTGSLLSLSEQELIDcDR(S)	100%	129.15	(+57)	1,075.54	2	0.0003228
(K)ITTGSLLSLSEQELIDcDR(S)	100%	25.55	(+57)	717.36	3	0.0004918
(R)SYNTGcGGGLmTYAYK(F)	100%	71.16	(+57), (+16)	879.8762	2	-0.001282
(K)NGGIDTEDDYPFR(E)	100%	85.24		749.8257	2	-0.0006539
(K)NGGIDTEDDYPFREADGTcNK(N)	97%	16.48	(+57)	792.0014	3	0.0004488
(K)KHVVTIDGYK(E)	56%	13.52		580.3271	2	-0.0002479
(K)HVVTIDGYK(E)	100%	46.37		516.2797	2	0.00002606
(K)HVVTIDGYK(E)	100%	42.99		516.2795	2	-0.0003399
(K)HVVTIDGYK(E)	58%	17.94		516.2802	2	0.001002
(R)NTGSSSGIcGINMmASFPTK(T)	100%	67.87	(+57), (+16)	1,038.46	2	0.00009975
(R)NTGSSSGIcGINmmASFPTK(T)	100%	54.2	(+57), (+16), (+16)	1,046.46	2	-0.003359
(R)NTGSSSGIcGINmmASFPTK(T)	95%	23.81	(+57), (+16), (+16)	697.9748	3	-0.004717
(R)SccPHDYPIcDTAR(G)	100%	41.49	(+57), (+57), (+57)	584.569	3	-0.001284

Table S5. Peptide sequences of *Hv*Pap-12 (P05167) with 9 exclusive unique peptides, 13 exclusive unique spectra, 83 total spectra, and 26% coverage (162/366 amino acids). The sample (representative of three experiments) was excised from the 38-kDa band of SDS-PAGE.

Peptide Sequence	Probability	Ion Score	Modifications	Observed m/z	Charge	Delta Da
(R)DAAALPETK(D)	100%	45.93		458.2425	2	-0.00058
(R)DAAALPETK(D)	100%	39.62		458.2429	2	0.000156
(R)DAAALPETK(D)	100%	34.68		458.2427	2	-0.00021
(R)DAAALPETK(D)	100%	33.83		458.2427	2	-0.00015
(R)DAAALPETK(D)	99%	25.27		458.2428	2	3.41E-05
(R)DAAALPETK(D)	90%	19.4		458.2426	2	-0.00051
(R)DAAALPETK(D)	72%	15.77		458.2429	2	0.000156
(R)DAAALPETK(D)	59%	14.51		458.2425	2	-0.00058
(R)DAAALPETK(D)	41%	12.13		458.2429	2	9.61E-05
(R)DAAALPETKDWREDGIVSPVK(N)	61%	11.32		766.3977	3	0.001017
(K)DWREDGIVSPVK(N)	100%	46.67		467.5776	3	0.00122
(K)DWREDGIVSPVK(N)	100%	42.14		467.5771	3	-0.00015
(K)DWREDGIVSPVK(N)	100%	40.81		467.5771	3	-0.00034
(K)DWREDGIVSPVK(N)	100%	40.85		467.5772	3	-6.1E-05
(K)DWREDGIVSPVK(N)	100%	40.85		467.5771	3	-0.00043
(K)DWREDGIVSPVK(N)	100%	40.49		467.577	3	-0.00061
(K)DWREDGIVSPVK(N)	100%	35.79		467.5771	3	-0.00024
(K)DWREDGIVSPVK(N)	100%	36.09		467.5771	3	-0.00043
(K)DWREDGIVSPVK(N)	100%	53.52		700.8628	2	0.001264
(K)DWREDGIVSPVK(N)	100%	34.06		467.577	3	-0.00052
(K)DWREDGIVSPVK(N)	100%	47.28		700.862	2	-0.00032
(K)DWREDGIVSPVK(N)	100%	29.52		467.5771	3	-0.00043
(K)DWREDGIVSPVK(N)	100%	35.85		700.862	2	-0.00032
(K)DWREDGIVSPVK(N)	100%	25.1		467.5775	3	0.000764
(K)DWREDGIVSPVK(N)	100%	32.27		700.8624	2	0.000532
(K)DWREDGIVSPVK(N)	98%	19.73		467.577	3	-0.00061
(K)DWREDGIVSPVK(N)	98%	24.56		700.8619	2	-0.00045
(K)DWREDGIVSPVK(N)	97%	23.38		700.8621	2	-0.0002
(K)DWREDGIVSPVK(N)	95%	21.4		700.8619	2	-0.00057
(K)DWREDGIVSPVK(N)	90%	19.2		700.8619	2	-0.00057
(K)DWREDGIVSPVK(N)	62%	10.43		467.5771	3	-0.00034
(K)DWREDGIVSPVK(N)	18%	6.68		467.5772	3	0.000122
(R)EDGIVSPVK(N)	100%	44.74		472.2583	2	-0.00026
(R)EDGIVSPVK(N)	100%	43.75		472.2584	2	-0.00014
(R)EDGIVSPVK(N)	100%	46.4		472.2588	2	0.000718
(R)EDGIVSPVK(N)	100%	34.1		472.2583	2	-0.00032

(R)EDGIVSPVK(N)	100%	34.09		472.2583	2	-0.00032
(R)EDGIVSPVK(N)	100%	31.24		472.2582	2	-0.00056
(R)EDGIVSPVK(N)	45%	12.34		472.2584	2	-0.0002
(R)EDGIVSPVK(N)	8%	6.53		472.2584	2	-0.00014
(R)EDGIVSPVK(N)	6%	6.24		472.2586	2	0.00029
(K)YNGGIDTEESYPYK(G)	100%	91.24		818.3622	2	-0.00023
(K)YNGGIDTEESYPYK(G)	100%	91.14		818.3622	2	-0.00036
(K)YNGGIDTEESYPYK(G)	100%	89.67		818.3621	2	-0.00048
(K)YNGGIDTEESYPYK(G)	100%	76.38		818.3624	2	1.01E-05
(K)YNGGIDTEESYPYK(G)	100%	70.73		818.3622	2	-0.00023
(K)YNGGIDTEESYPYK(G)	100%	58.71		818.3624	2	0.000132
(K)YNGGIDTEESYPYK(G)	100%	59.14		818.3623	2	-0.00011
(K)YNGGIDTEESYPYK(G)	100%	54.28		818.3622	2	-0.00023
(K)YNGGIDTEESYPYK(G)	100%	55.91		818.3609	2	-0.00292
(K)YNGGIDTEESYPYK(G)	100%	26.15		545.9107	3	0.00012
(K)YNGGIDTEESYPYK(G)	100%	29.55		818.3619	2	-0.00085
(K)YNGGIDTEESYPYK(G)	99%	36.47		818.3611	2	-0.00255
(K)YNGGIDTEESYPYK(G)	93%	20.18		818.3623	2	-0.00011
(K)GVNGVcHYK(A)	100%	36.81	(+57)	517.2475	2	-0.00069
(K)NAVGLVRPVSVAFQVIDGFR(Q)	100%	48.29		715.4041	3	6.11E-05
(K)NAVGLVRPVSVAFQVIDGFR(Q)	100%	47.34		715.4041	3	0.000244
(K)NAVGLVRPVSVAFQVIDGFR(Q)	100%	44.91		715.4044	3	0.001159
(K)NAVGLVRPVSVAFQVIDGFR(Q)	89%	18.92		715.4033	3	-0.00232
(K)NSWGADWGDNGYFK(M)	100%	80.79		808.8419	2	-0.00011
(K)NSWGADWGDNGYFK(M)	100%	55.92		808.8423	2	0.00087
(K)NSWGADWGDNGYFK(M)	100%	51.77		808.8425	2	0.001114
(K)NSWGADWGDNGYFK(M)	100%	44.02		808.8418	2	-0.00023
(K)NSWGADWGDNGYFK(M)	92%	24.49		808.8427	2	0.00148
(K)NSWGADWGDNGYFK(M)	28%	10.58		808.8415	2	-0.00084
(K)NSWGADWGDNGYFK(M)	25%	12.65		808.8425	2	0.001114
(K)NMcAIATcASYPVVAA(-)	100%	92.37	(+57), (+57)	849.8859	2	-0.00032
(K)NMcAIATcASYPVVAA(-)	100%	77.21	(+57), (+57)	849.8857	2	-0.00069
(K)NmcAIATcASYPVVAA(-)	100%	31.41	(+16), (+57), (+57)	572.258	3	-0.00026
(K)NMcAIATcASYPVVAA(-)	100%	43.61	(+57), (+57)	849.8859	2	-0.00032
(K)NmcAIATcASYPVVAA(-)	100%	41.56	(+16), (+57), (+57)	857.8832	2	-0.00061
(K)NmcAIATcASYPVVAA(-)	100%	34.64	(+16), (+57), (+57)	857.8835	2	4.47E-07
(K)NMcAIATcASYPVVAA(-)	100%	34.16	(+57), (+57)	849.8858	2	-0.00045
(K)NMcAIATcASYPVVAA(-)	100%	31.44	(+57), (+57)	849.8857	2	-0.00057
(K)NMcAIATcASYPVVAA(-)	99%	27.82	(+57), (+57)	849.8855	2	-0.00106
(K)NMcAIATcASYPVVAA(-)	98%	24.93	(+57), (+57)	849.8856	2	-0.00081
(K)NMcAIATcASYPVVAA(-)	98%	29.31	(+57), (+57)	849.8849	2	-0.00228

(K)NMcAIATcASYPVVAA(-)	97%	26.2	(+57), (+57)	849.8866	2	0.001141
(K)NMcAIATcASYPVVAA(-)	92%	21.22	(+57), (+57)	849.8854	2	-0.0013
(K)NMcAIATcASYPVVAA(-)	88%	20.25	(+57), (+57)	849.8853	2	-0.00155
(K)NMcAIATcASYPVVAA(-)	82%	17.73	(+57), (+57)	849.8855	2	-0.00106
(K)NMcAIATcASYPVVAA(-)	25%	9.8	(+57), (+57)	849.8859	2	-0.0002
(K)NMcAIATcASYPVVAA(-)	18%	9.05	(+57), (+57)	849.8857	2	-0.00069

Table S6. Peptide sequences of *Hv*Pap-13 (A0A8I6XJP4) with 13 exclusive unique peptides, 19 exclusive unique spectra, 40 total spectra, and 44% coverage (162/366 amino acids). The sample (representative of three experiments) was excised from the 38-kDa band of SDS-PAGE.

Peptide Sequence	Probability	Ion Score	Modifications	Observed m/z	Charge	Delta Da
(K)LASLSEQELVDcDK(L)	100%	89.29	(+57)	803.8851	2	-0.0001232
(K)LASLSEQELVDcDKLDHGcNGGVSYR(A)	70%	18.69	(+57), (+57)	974.7817	3	-0.005498
(K)LASLSEQELVDcDKLDHGcNGGVSYR(A)	9%	5.06	(+57), (+57)	731.3392	4	-0.001117
(K)LDHGcNGGVSYR(A)	31%	8.05	(+57)	445.5351	3	0.00002176
(R)ALQWITSNGGITSQDDYPYTAK(D)	100%	79.69		1,215.08	2	-0.0008199
(R)ALQWITSNGGITSQDDYPYTAK(D)	100%	60.11		810.3924	3	0.0003871
(R)ALQWITSNGGITSQDDYPYTAKDDTcDTTK(L)	100%	61.28	(+57)	1,122.51	3	-0.002758
(K)LSHHAASISGFQR(V)	100%	67.55		470.9127	3	-0.0001309
(K)LSHHAASISGFQR(V)	100%	57.84		470.9132	3	0.001426
(K)LSHHAASISGFQR(V)	100%	39.88		470.9127	3	-0.0001309
(K)LSHHAASISGFQR(V)	100%	42.52		705.8662	2	0.0015
(K)LSHHAASISGFQR(V)	100%	23.84		470.9128	3	0.0002351
(K)LSHHAASISGFQR(V)	100%	29.71		705.8655	2	0.00003606
(K)LSHHAASISGFQR(V)	99%	20.55		470.9125	3	-0.0005869
(K)LSHHAASISGFQR(V)	74%	11.53		470.9126	3	-0.0003139
(R)SELSLTNAVAmQPVAVSIEAGGANFQHYR(N)	100%	23.23	(+16)	1,026.18	3	0.000427
(R)NGVYNGPcGTR(L)	100%	33.74	(+57)	597.7692	2	-0.001057
(R)NGVYNGPcGTR(L)	91%	19.73	(+57)	597.7698	2	0.0001628
(R)LNHGVTVVGYGK(D)	100%	70.31		622.3432	2	-0.0003199
(R)LNHGVTVVGYGK(D)	100%	46.53		415.2312	3	-0.0003019
(R)LNHGVTVVGYGK(D)	100%	29.13		415.2313	3	-0.0002119
(R)LNHGVTVVGYGKDEVTR(E)	100%	89.21		615.3268	3	-0.0004519
(R)LNHGVTVVGYGKDEVTR(E)	100%	66.13		615.3269	3	-0.00008594
(R)LNHGVTVVGYGKDEVTR(E)	100%	59.79		461.747	4	0.00008606
(R)LNHGVTVVGYGKDEVTR(E)	100%	71.67		922.4868	2	-0.00001194
(R)LNHGVTVVGYGKDEVTR(E)	100%	39.58		461.747	4	-0.0001579
(R)LNHGVTVVGYGKDEVTR(E)	100%	38.31		615.3268	3	-0.0002689
(R)LNHGVTVVGYGKDEVTR(E)	100%	31.83		461.7469	4	-0.0002819
(R)LNHGVTVVGYGKDEVTR(E)	100%	30.06		615.3273	3	0.001015

(R)LNHGVTVVGYGKDEVTR(E)	100%	25.41		461.7469	4	-0.0002819
(R)LNHGVTVVGYGKDEVTR(E)	96%	21.34		922.4868	2	-0.00001194
(R)LNHGVTVVGYGKDEVTR(E)	74%	11.5		461.747	4	-0.00003794
(R)ESYWIVK(N)	90%	28.25		462.745	2	0.0001681
(R)ESYWIVK(N)	83%	19.07		462.7451	2	0.0004121
(R)ESYWIVK(N)	82%	17.31		462.745	2	0.0001081
(R)ESYWIVK(N)	80%	16.81		462.7448	2	-0.0001359
(R)ESYWIVK(N)	24%	10.08		462.745	2	0.0001081
(K)WGDNGYLR(M)	99%	31.52		490.7328	2	-0.0002459
(K)WGDNGYLR(M)	42%	11.97		490.7328	2	-0.0001839
(K)GIIDKPEGIcGIAIRPSFPLV(-)	100%	29.19	(+57)	751.4211	3	0.001083

Table S7. Peptide sequences of *Hv*Pap-14 (B4ESF2) with 12 exclusive unique peptides, 27 exclusive unique spectra, 73 total spectra, and 37% coverage (134/367 amino acids). The sample (representative of three experiments) was excised from the 38-kDa band of SDS-PAGE.

**Peptide Sequence**	**Probability**	**Ion Score**	**Modifications**	**Observed m/z**	**Charge**	**Delta Da**
(K)cGTELDHGVAAVGYGTTVDGTK(Y)	12%	6.03	(+57)	736.6797	3	0.0005838
(K)cGTELDHGVAAVGYGTTVDGTK(Y)	100%	41.43	(+57)	736.6791	3	-0.001249
(K)cGTELDHGVAAVGYGTTVDGTK(Y)	100%	48.02	(+57)	736.6796	3	0.00003176
(K)cGTELDHGVAAVGYGTTVDGTK(Y)	100%	53.36	(+57)	736.6796	3	0.00003176
(K)cGTELDHGVAAVGYGTTVDGTK(Y)	100%	56.29	(+57)	736.6792	3	-0.001066
(K)cGTELDHGVAAVGYGTTVDGTK(Y)	100%	97.14	(+57)	1,104.52	2	0.0001068
(K)DKEGLcGIAMEASYPVK(T)	97%	16.92	(+57)	623.3026	3	-0.00001024
(K)DKEGLcGIAmEASYPVK(T)	100%	35.71	(+57), (+16)	942.4479	2	0.0004497
(K)DKEGLcGIAmEASYPVK(T)	100%	37.14	(+57), (+16)	942.4479	2	0.0004497
(K)DKEGLcGIAMEASYPVK(T)	100%	35.38	(+57)	623.3032	3	0.002006
(K)DKEGLcGIAmEASYPVK(T)	100%	31.51	(+57), (+16)	628.634	3	-0.0004793
(K)DKEGLcGIAMEASYPVK(T)	100%	49.72	(+57)	934.45	2	-0.0003632
(K)DKEGLcGIAmEASYPVK(T)	100%	44.8	(+57), (+16)	628.6341	3	-0.0002963
(R)DVKDKEGLcGIAMEASYPVK(T)	100%	23.09	(+57)	553.2765	4	0.0006168
(R)DVKDKEGLcGIAMEASYPVK(T)	100%	33.32	(+57)	737.3662	3	0.0005698
(R)DVKDKEGLcGIAmEASYPVK(T)	100%	37.08	(+57), (+16)	742.6978	3	0.0004667
(R)DVPPSVDWR(Q)	14%	7.92		535.7669	2	-0.0001079
(R)DVPPSVDWR(Q)	76%	17.28		535.7672	2	0.0003801
(R)DVPPSVDWR(Q)	78%	16.37		535.7668	2	-0.0002299
(R)DVPPSVDWR(Q)	80%	16.96		535.767	2	0.0001361
(R)DVPPSVDWR(Q)	88%	18.54		535.7668	2	-0.0003519
(R)DVPPSVDWR(Q)	89%	18.96		535.7668	2	-0.0002299
(R)DVPPSVDWR(Q)	90%	18.88		535.7669	2	-0.0001079
(R)DVPPSVDWR(Q)	91%	19.1		535.7669	2	-0.0001079

(R)DVPPSVDWR(Q)	91%	19.3		535.7669	2	-0.0001079
(R)DVPPSVDWR(Q)	91%	18.47		535.767	2	0.00001406
(R)DVPPSVDWR(Q)	91%	19.63		535.7668	2	-0.0002299
(R)DVPPSVDWR(Q)	94%	19.51		535.767	2	0.00001406
(R)DVPPSVDWR(Q)	94%	19.74		535.767	2	0.00001406
(K)EGGGGFmHGSAASVR(D)	96%	16.46	(+16)	479.2175	3	-0.000509
(K)EGLcGIAmEASYPVK(T)	7%	6.2	(+57), (+16)	820.8862	2	-0.0008423
(K)EGLcGIAmEASYPVK(T)	99%	19.77	(+57), (+16)	547.5936	3	0.0003057
(K)EGLcGIAMEASYPVK(T)	100%	41.53	(+57)	812.8889	2	-0.0006772
(K)EGLcGIAMEASYPVK(T)	100%	41.81	(+57)	812.8889	2	-0.0006772
(K)EGLcGIAmEASYPVK(T)	100%	69.86	(+57), (+16)	820.8864	2	-0.0004743
(K)HGGVAAEDAYPYK(A)	17%	6.57		459.886	3	0.0001431
(K)HGGVAAEDAYPYK(A)	55%	13.64		689.3251	2	-0.0005759
(K)HGGVAAEDAYPYK(A)	82%	18.35		689.3256	2	0.0005241
(K)HGGVAAEDAYPYK(A)	89%	13.76		459.886	3	-0.00003994
(K)HGGVAAEDAYPYK(A)	95%	16.22		459.8859	3	-0.0004059
(K)HGGVAAEDAYPYK(A)	96%	16.38		459.886	3	-0.00003994
(K)HGGVAAEDAYPYK(A)	100%	21.08		459.886	3	0.00005306
(K)HGGVAAEDAYPYK(A)	100%	46.85		459.8859	3	-0.0003129
(K)HGGVAAEDAYPYK(A)	100%	51.49		459.886	3	-0.00003994
(K)HGGVAAEDAYPYK(A)	100%	70.17		689.3252	2	-0.0003319
(K)HGGVAAEDAYPYK(A)	100%	67.95		459.8859	3	-0.0002229
(K)HGGVAAEDAYPYK(A)	100%	85.13		689.3253	2	-0.0002099
(K)HGGVAAEDAYPYK(A)	100%	88.48		689.3254	2	0.00003406
(K)HGGVAAEDAYPYKAR(Q)	22%	8.49		535.5985	3	-0.0008479
(K)KPSAVVTIDGYEDVPANDETALK(K)	17%	11.23		811.4105	3	-0.002615
(K)KPSAVVTIDGYEDVPANDETALK(K)	100%	50.64		811.4108	3	-0.001697
(K)KPSAVVTIDGYEDVPANDETALK(K)	100%	70.88		1,216.61	2	-0.0004039
(K)KPSAVVTIDGYEDVPANDETALK(K)	100%	57.31		811.411	3	-0.0009649
(K)KPSAVVTIDGYEDVPANDETALKK(A)	30%	13.06		854.1085	3	-0.003435
(K)KPSAVVTIDGYEDVPANDETALKK(A)	87%	13.24		640.8342	4	0.0003941
(K)KPSAVVTIDGYEDVPANDETALKK(A)	94%	37.01		512.8687	5	0.00001506
(K)KPSAVVTIDGYEDVPANDETALKK(A)	98%	29.54		1,280.66	2	-0.003118
(K)KPSAVVTIDGYEDVPANDETALKK(A)	100%	29.32		854.1097	3	0.00004206
(K)KPSAVVTIDGYEDVPANDETALKK(A)	100%	41.27		640.8344	4	0.001374
(K)KPSAVVTIDGYEDVPANDETALKK(A)	100%	49.79		640.8336	4	-0.001802
(K)KPSAVVTIDGYEDVPANDETALKK(A)	100%	48.52		640.8341	4	0.0001501
(K)KPSAVVTIDGYEDVPANDETALKK(A)	100%	57.33		640.8341	4	0.0001501
(K)KPSAVVTIDGYEDVPANDETALKK(A)	100%	80.09		854.1097	3	0.00004206
(K)KPSAVVTIDGYEDVPANDETALKK(A)	100%	97.35		854.1097	3	0.00004206
(K)NSWGPEWGEK(G)	16%	8.73		595.2674	2	0.0001681

(K)NSWGPEWGEK(G)	73%	17.34		595.2676	2	0.0005341
(K)NSWGPEWGEK(G)	98%	24.14		595.2673	2	0.00004606
(K)NSWGPEWGEK(G)	100%	35.06		595.2672	2	-0.0003199
(K)SNAGcNGGLMDYAFQYIAK(H)	35%	18.64	(+57)	1,040.47	2	-0.003755
(K)SNAGcNGGLmDYAFQYIAK(H)	100%	27.1	(+57), (+16)	699.3116	3	-0.0009403
(K)SNAGcNGGLmDYAFQYIAK(H)	100%	27.87	(+57), (+16)	699.3116	3	-0.0009403
(K)SNAGcNGGLmDYAFQYIAK(H)	100%	38.93	(+57), (+16)	1,048.46	2	-0.001112
(K)SNAGcNGGLmDYAFQYIAK(H)	100%	65.14	(+57), (+16)	1,048.46	2	0.001086

Table S8. Peptide sequences of *Hv*Pap-16 (B4ESF4) with 9 exclusive unique peptides, 10 exclusive unique spectra, 10 total spectra, and 21% coverage (83/389 amino acids). The sample (representative of three experiments) was excised from the 38-kDa band of SDS-PAGE.

**Peptide Sequence**	**Probability**	**Ion Score**	**Modifications**	**Observed m/z**	**Charge**	**Delta Da**
(R)GWmDDAFK(W)	12%	6.62	(+16)	493.2079	2	-0.00000495
(K)AGNcQTGKPVAVR(L)	100%	23.42	(+57)	453.2384	3	0.00004576
(K)KVTPPGNEAGLK(E)	97%	25.3		404.2303	3	-0.002757
(K)VTPPGNEAGLK(E)	61%	14.23		541.7956	2	-0.0003359
(K)TAQNHAmALVGYGTKPDGTK(Y)	100%	29.14	(+16)	692.6773	3	-0.000792
(K)TAQNHAmALVGYGTKPDGTK(Y)	36%	8.37	(+16)	519.7597	4	-0.001169
(K)WGDKGFIYLLR(D)	37%	8.61		456.5873	3	-0.00004794
(K)GFIYLLR(D)	90%	32.7		441.2658	2	-0.0001059
(R)DSPPLGLcGLAK(L)	84%	18.72	(+57)	614.3234	2	-0.0009372
(K)LPVYPII(-)	77%	16.38		407.7572	2	-0.0002559

Table S9. Peptide sequences of *Hv*Pap-17 (A0A8I6Y6A5) with 6 exclusive unique peptides, 7 exclusive unique spectra, 12 total spectra, and 21% coverage (73/349 amino acids). The sample (representative of three experiments) was excised from the 38-kDa band of SDS-PAGE.

**Peptide Sequence**	**Probability**	**Ion Score**	**Modifications**	**Observed m/z**	**Charge**	**Delta Da**
(R)RPPAAAAGAGSGAGR(F)	92%	14.51		422.8936	3	-0.0001329
(R)AmGAVTGVK(D)	100%	64.41	(+16)	425.2286	2	-0.00008295
(R)AMGAVTGVK(D)	100%	42.97		417.2311	2	-0.0001639
(R)GGLTTESSYPYR(G)	100%	57.23		665.8173	2	-0.00007794
(R)GGLTTESSYPYR(G)	100%	56.42		665.8171	2	-0.0005659
(R)GGLTTESSYPYR(G)	92%	18.85		665.8174	2	0.00004406
(R)GGLTTESSYPYR(G)	6%	6.89		665.8176	2	0.0005321
(R)SASAASIR(G)	99%	37.72		381.7089	2	0.0001021
(K)NSWGGSWGEGGYVR(I)	100%	58.61		756.3361	2	-0.00128
(K)NSWGGSWGEGGYVR(I)	100%	31.49		756.3365	2	-0.0005479
(K)NSWGGSWGEGGYVR(I)	58%	12.83		756.3368	2	0.00006206

(R)GEGVcGLAQLASYPV(-)	100%	62.04	(+57)	760.8744	2	-0.000007243

Table S10. Peptide sequences of *Hv*Pap-19 (A0A8I7BBL8) with 13 exclusive unique peptides, 25 exclusive unique spectra, 40 total spectra, and 33% coverage (112/344 amino acids). The sample (representative of three experiments) was excised from the 38-kDa band of SDS-PAGE.

Peptide Sequence	Probability	Ion Score	Modifications	Observed m/z	Charge	Delta Da
(R)SEQLPKEFDAR(S)	97%	18.56		440.5577	3	-0.0005709
(R)SKWSGcSTIGK(I)	100%	53.32	(+57)	404.201	3	-0.0002892
(R)SKWSGcSTIGK(I)	96%	22.48	(+57)	605.7978	2	-0.0003052
(K)WSGcSTIGK(I)	100%	59.01	(+57)	498.2342	2	-0.0004432
(K)HPGcEPAYPTPVcEK(K)	100%	39.29	(+57), (+57)	581.2605	3	-0.0005345
(K)HPGcEPAYPTPVcEK(K)	100%	36.8	(+57), (+57)	581.2604	3	-0.0009005
(K)HPGcEPAYPTPVcEK(K)	66%	14.78	(+57), (+57)	871.3871	2	-0.0004605
(K)HPGcEPAYPTPVcEK(K)	47%	12.85	(+57), (+57)	871.3869	2	-0.0009485
(K)cKVQNQVWQEK(K)	98%	17.73	(+57)	482.9101	3	-0.00008524
(K)cKVQNQVWQEK(K)	86%	18	(+57)	723.8615	2	-0.0002872
(K)VQNQVWQEK(K)	100%	48.55		579.7985	2	-0.0005979
(K)VQNQVWQEK(K)	97%	24.09		579.7984	2	-0.0007199
(K)VQNQVWQEK(K)	45%	13.91		579.799	2	0.0005001
(K)VQNQVWQEKK(H)	100%	37.89		429.5666	3	-0.00003094
(K)VQNQVWQEKK(H)	100%	37.56		643.8463	2	-0.00001794
(K)KHFSIDAYQVNSDPHDImAEVYK(N)	46%	9.21	(+16)	681.5748	4	0.00008305
(K)HFSIDAYQVNSDPHDImAEVYK(N)	100%	40.62	(+16)	649.5513	4	0.001091
(K)HFSIDAYQVNSDPHDImAEVYK(N)	100%	39.21	(+16)	865.7322	3	-0.000179
(K)HFSIDAYQVNSDPHDImAEVYK(N)	100%	33.77	(+16)	649.5507	4	-0.001109
(K)HFSIDAYQVNSDPHDImAEVYK(N)	95%	19.64	(+16)	865.7314	3	-0.002378
(K)HITGGVMGGHAVK(L)	100%	66.17		421.8924	3	-0.0001199
(K)HITGGVmGGHAVK(L)	100%	48.84	(+16)	427.2239	3	-0.000499
(K)HITGGVmGGHAVK(L)	100%	41.29	(+16)	427.2239	3	-0.000592
(K)HITGGVmGGHAVK(L)	100%	40.95	(+16)	640.3323	2	-0.000487
(K)HITGGVMGGHAVK(L)	100%	37.13		632.335	2	-0.00007794
(K)HITGGVMGGHAVK(L)	99%	20.86		421.8923	3	-0.0003959
(K)HITGGVmGGHAVK(L)	98%	24.3	(+16)	640.3322	2	-0.000609
(K)HITGGVMGGHAVK(L)	46%	9.17		421.8924	3	-0.0003029
(R)GWGDDGYFK(I)	100%	34.56		522.7247	2	-0.0002819
(R)GWGDDGYFK(I)	76%	16.26		522.7247	2	-0.0002819
(R)GWGDDGYFK(I)	59%	13.92		522.7247	2	-0.0001599
(R)GWGDDGYFK(I)	26%	10.74		522.7245	2	-0.0006479
(R)GWGDDGYFK(I)	23%	9.48		522.7248	2	-0.00003794

(R)GKNEcGIEEDVTAGmPSmK(N)	100%	69.71	(+57), (+16), (+16)	695.6356	3	-0.001516
(R)GKNEcGIEEDVTAGmPSmK(N)	100%	44.65	(+57), (+16), (+16)	1,042.95	2	-0.0002833
(R)GKNEcGIEEDVTAGmPSmK(N)	99%	18.64	(+57), (+16), (+16)	695.6361	3	0.0001307
(R)GKNEcGIEEDVTAGMPSMK(N)	96%	16.05	(+57)	684.9727	3	-0.0002122
(R)GKNEcGIEEDVTAGMPSmK(N)	90%	13.87	(+57), (+16)	690.3043	3	-0.0004983
(R)GKNEcGIEEDVTAGmPSMK(N)	24%	15.03	(+57), (+16)	1,034.95	2	-0.002926
(K)NEcGIEEDVTAGmPSmK(N)	100%	82.03	(+57), (+16), (+16)	950.3914	2	-0.001581

Table S11. Peptide sequences of *Hv*Pap-20 (A0A8I7B6S7) with 3 exclusive unique peptides, 3 exclusive unique spectra, 17 total spectra, and 18% coverage (62/353 amino acids). The sample (representative of three experiments) was excised from the 38-kDa band of SDS-PAGE.

Peptide Sequence	Probability	Ion Score	Modifications	Observed m/z	Charge	Delta Da
(K)VENQVWK(K)	90%	37.31		451.7402	2	0.00002406
(R)VHSNPHDImAEVYK(N)	100%	32.11	(+16)	552.5998	3	0.000163
(R)VHSNPHDImAEVYK(N)	94%	15.91	(+16)	552.6	3	0.000715
(K)HITGGVMGGHAVK(L)	100%	66.17		421.8924	3	-0.0001199
(K)HITGGVmGGHAVK(L)	100%	48.84	(+16)	427.2239	3	-0.000499
(K)HITGGVmGGHAVK(L)	100%	41.29	(+16)	427.2239	3	-0.000592
(K)HITGGVmGGHAVK(L)	100%	40.95	(+16)	640.3323	2	-0.000487
(K)HITGGVMGGHAVK(L)	100%	37.13		632.335	2	-0.00007794
(K)HITGGVMGGHAVK(L)	99%	20.86		421.8923	3	-0.0003959
(K)HITGGVmGGHAVK(L)	98%	24.3	(+16)	640.3322	2	-0.000609
(K)HITGGVMGGHAVK(L)	46%	9.17		421.8924	3	-0.0003029
(R)GWGDDGYFK(I)	100%	34.56		522.7247	2	-0.0002819
(R)GWGDDGYFK(I)	76%	16.26		522.7247	2	-0.0002819
(R)GWGDDGYFK(I)	59%	13.92		522.7247	2	-0.0001599
(R)GWGDDGYFK(I)	26%	10.74		522.7245	2	-0.0006479
(R)GWGDDGYFK(I)	23%	9.48		522.7248	2	-0.00003794
(R)GKNEcGIEEDVTAGmPSTK(N)	100%	30.58	(+57), (+16)	680.3069	3	0.0001767

Supplementary Table S12. Distribution of barley L-like E cathepsin orthologs across plant species

Species	Number of Orthologs	Plant Family	Clade	Plant Subfamily	Pathway
Aegilops tauschii (Tausch's goatgrass)	7	Poaceae	BOP	Pooideae	C3
Aegilops tauschii subsp. strangulata	22	Poaceae	BOP	Pooideae	C3
Brachypodium distachyon	9	Poaceae	BOP	Pooideae	C3
Eragrostis curvula (weeping love grass)	1	Poaceae	PACMAD	Chloridoideae	C4
Leersia perrieri	3	Poaceae	BOP	Oryzoideae	C3
Lolium multiflorum (Italian ryegrass)	11	Poaceae	BOP	Pooideae	C3
Oryza barthii	2	Poaceae	BOP	Ehrhartoideae	C3
Oryza brachyantha (malo sina)	2	Poaceae	BOP	Ehrhartoideae	C3
Oryza glaberrima (African rice)	3	Poaceae	BOP	Ehrhartoideae	C3
Oryza glumipatula	8	Poaceae	BOP	Ehrhartoideae	C3
Oryza nivara (Indian wild rice)	5	Poaceae	BOP	Oryzoideae	C3
Oryza punctata (Red rice)	4	Poaceae	BOP	Oryzoideae	C3
Oryza rufipogon (Brownbeard rice)	4	Poaceae	BOP	Ehrhartoideae	C3
Oryza sativa (Rice)	20	Poaceae	BOP	Ehrhartoideae	C3
Paspalum notatum var. saurae	1	Poaceae	PACMAD	Panicoideae	C4
Paspalum vaginatum	2	Poaceae	PACMAD	Panicoideae	C4
Setaria italica (Foxtail millet)	6	Poaceae	PACMAD	Panicoideae	C4
Setaria viridis (Green bristlegrass)	4	Poaceae	PACMAD	Panicoideae	C4
Triticum aestivum	113	Poaceae	BOP	Pooideae	C3
Triticum turgidum subsp. durum	48	Poaceae	BOP	Pooideae	C3
Triticum urartu	30	Poaceae	BOP	Pooideae	C3
Urochloa decumbens	13	Poaceae	PACMAD	Panicoideae	C4
Zingiber officinale (Ginger)	1	Zingiberaceae		Zingiberoideae	
Zizania palustris (Northern wild rice)	7	Poaceae	BOP	Oryzoideae	C3
Sorghum vulgare (Sorghum)	0	Poaceae	PACMAD	Panicoideae	C4
Zea mays (Maize)	0	Poaceae	PACMAD	Panicoideae	C4
**Total number of orthologs**	**326**				

Amino acid sequences with up to 50% sequence similarity to eighteen cathepsin L-like E barley PLCPs (*Hv*Pap-13, -15, -16, -18, -21, -22, -25, -27, -28, -29, -33, -34, -35, -36, -37, -38; A0A8I6XNC2, and F2CPT6) were retrieved from the UniProt open-access resource. PACMAD clade abbreviation derived from the first letters of the subfamilies it contains: Panicoideae, Arundinoideae, Chloridoideae, Micrairoideae, Aristidoideae, and Danthonioideae; BOP clade stands for Bambusoideae, Oryzoideae, and Pooideae.
